# Photocatalytic Recovery of Aromatic Chemicals from Waste Plastics

**DOI:** 10.1002/advs.77184

**Published:** 2026-08-15

**Authors:** Lizhen Liu, Jutarat Jitrada, Jun Huang

**Affiliations:** ^1^ Laboratory for Catalysis Engineering School of Chemical and Biomolecular Engineering The University of Sydney Sydney New South Wales Australia

**Keywords:** aromatic chemicals, photocatalysis, photosynthesis, plastic upcycling, waste plastics

## Abstract

Plastic waste is abundant in carbon and hydrogen resources. The development of sustainable catalytic process for the recovery of chemicals from waste plastic offers a promising strategy to simultaneously mitigate environmental pollution and reduce dependence on petrochemical products. Among the valuable targets, aromatic chemicals are particularly attractive which are widely used in polymers, pharmaceuticals, and fuels. Therefore, photocatalytic recovery of aromatic chemicals from waste plastic attracts huge attention to contribute to sustainable technology and circular economy, which couple solar energy with catalyst‐controlled redox chemistry to enable plastic upcycling under mild conditions. In this Review, the fundamentals of photocatalytic plastic upcycling are outlined, including photocatalytic mechanism and the thermodynamic and kinetic challenges. Then, representative catalytic systems are discussed, such as polyethylene terephthalate, polystyrene, and polyphenylene sulfide. Finally, the future challenges and development directions are summarized, including development of catalytic system, mechanistic elucidation, real‐world plastic feedstocks, and techno‐economic analysis. This Review provides a comprehensive framework for understanding photocatalytic aromatic chemical recovery from waste plastic and offers guidance for the rational design of efficient, selective, and scalable solar‐driven upcycling systems.

## Graphical abstract

This Review summarizes recent advances in the photocatalytic recovery of aromatic chemicals from plastic waste, with emphasis on fundamental mechanism, representative catalytic systems, and future challenges and development directions, which is expected to provide in‐depth and valuable insights and references into the key role of sustainable catalysis in advancing waste management and the circular economy.

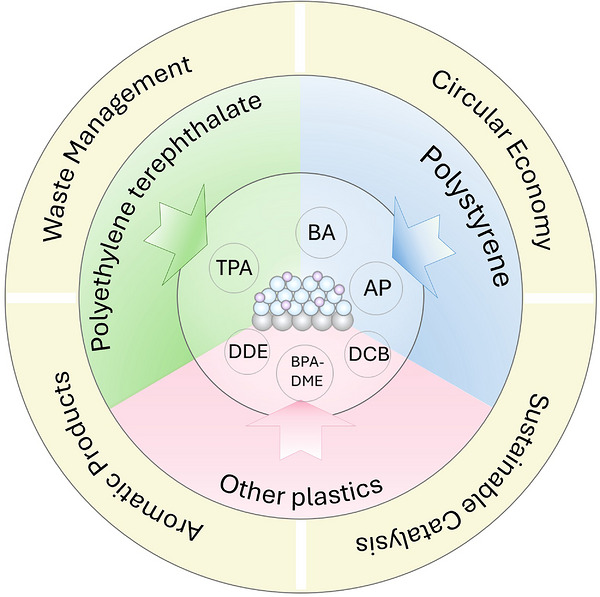

## 1 Introduction

The production and widespread use of plastics have brought enormous convenience to modern society, but they have also generated large volumes of plastic waste [1]. Over 70% of plastics are landfilled or incineration after use, making plastic pollution a world‐faced challenge [2, 3]. A sustainable society requires improved recovery of plastic waste. Conventional recycling routes include mechanical recycling and chemical recycling (pyrolysis and hydrogenolysis). Mechanical recycling typically involves physically shredding waste plastics and then sending them to a waste‐to‐energy plant, which results in downcycling and cause secondary pollution from microplastics [4, 5]. Pyrolysis and hydrogenolysis typically require substantial energy input, which can impose an additional energy burden and extra greenhouse gas emissions, imposing a substantial environmental burden and undermining the circular economy [6, 7, 8]. Therefore, the development of sustainable routes to recycle waste plastics is an urgent. Photocatalysis is regarded as new sustainable catalytic process since it was first proposed [9, 10]. It was widely applied in pollutant degradation, sustainable fuel production, and chemical valorization [11, 12]. Photocatalytic technology couples photons with catalyst‐controlled redox chemistry to deliver transformations at ambient pressure and moderate temperatures, avoiding the large thermal and hydrogen inputs demanded, which shows huge potential in plastic waste recycling. Photocatalysis can selectively depolymerize polymers and enable molecular‐level valorization to produce value‐added chemicals [13]. In addition, photocatalysis offers greater controllability over product selectivity and lower greenhouse gas emissions compared with pyrolysis and hydrogenolysis which often lead to non‐selective fragmentation and even mineralization [14, 15]. Previous studies reported plastic‐derived organics to produce fuels and chemicals via photoreforming and the direct catalytic oxidation of plastic into valuable chemicals [16, 17]. These advances highlight the promise of solar‐driven plastic conversion, with the potential to deliver sustainable and selective catalytic process that are difficult to achieve through conventional chemical recycling routes.

Among the valuable targets in plastic valorization, aromatic chemicals offer particularly high economic value. At present, aromatics are produced predominantly by the petrochemical industry and are widely used in polymers, pharmaceuticals, and fuels [18, 19, 20]. For example, benzoic acid (BA) and its salts are widely used as preservatives and chemical intermediates, while benzaldehyde is an important feedstock for flavor, fragrance, pharmaceutical, and agrochemical production. Terephthalic acid (TPA) is one of the most important monomers for polyester and polyethylene terephthalate (PET) manufacture. Styrene is a major building block for polystyrene (PS), synthetic rubber, insulation materials, packaging, and automotive components, whereas phenol is extensively used in the production of bisphenol A (BPA), phenolic resins, caprolactam, epoxy resins, and polycarbonate (PC) plastics. Therefore, recovering aromatic chemicals through the upcycling of waste plastics is of great significance for reducing dependence on fossil resources and advancing a circular economy. Aromatic structures are widely present in PET, PS, PC, polyphenylene sulfide (PPS), as well as styrenic copolymers [21, 22, 23]. These plastics account for over 15% of total plastic production, offering substantial opportunities for production of aromatic chemicals. Moreover, most of these plastics are non‐biodegradable, thus recycling of them can simultaneously produce valuable chemical feedstocks and mitigate the environmental issues. Photocatalytic upcycling of plastic can selectively activate C‐H and C‐C bonds and enable controlled oxygenation to depolymerize polymers while preserving and functionalizing aromatic structures to produce BTX (benzene, toluene, and xylenes), phenols, BA, or styrenic monomer, which provides a promising route to convert plastic into valuable chemicals and attracts consideration attention compared to degradation [24, 25].

Recent studies have demonstrated the feasibility of recovering aromatic chemicals from plastics via photocatalysis. For example, Li et al. reported a formate‐mediated, non‐hydrogen deoxygenative reduction strategy to upgrades PET into alkyl p‐methylbenzoates [26]. Building on CO_2_∙̄ mediated photoredox chemistry, the authors harness formate as a convenient reductant to enable selective C─O bond deoxygenation while retaining the aromatic ring. Notably, this work achieved a turnover number (TON) of up to 1550 for methyl p‐methylbenzoate (MMB) formation and was further demonstrated at the gram scale using a continuous‐flow photoreactor, underscoring its practical potential for scalable aromatic chemical recovery from PET waste. Xu et al. employed a porphyrin‐based porous organic polymer (PPOP) as the photocatalyst to achieve the depolymerization and oxidative conversion of PS in air, affording BA with a high yield (72%) and excellent selectivity (97%) [27]. The method uses ethyl acetate as a green solvent and air as the oxidant, enabling efficient valorization of PS and PS‐based plastic wastes into BA. Mechanistic studies indicate that both singlet oxygen (^1^O_2_) and radical species play crucial roles in the depolymerization and oxidative process. Although recent studies have demonstrated the upcycling of various plastics into aromatic chemicals and have provided substantial mechanistic insights, several challenges still remain to be addressed, such as, the inherent insolubility of many polymers and the associated mass‐transport limitations, the stability of aromatic frameworks during depolymerization, and the complexity of elucidating catalytic reaction pathways and the kinetics of reactive radical species (ROS) [28]. Therefore, an integrated and in‐depth understanding of both the advances and challenges in photocatalytic upcycling of plastics to aromatic chemicals is urgently required to inform rational catalyst and process design and to guide future research in this field.

Herein, this review provides an in‐depth insight and perspective on the photocatalytic upcycling of plastic waste to aromatic chemicals, with emphasis on the fundamentals, recent breakthroughs, and remaining challenges. We first introduce the mechanistic fundamentals of photocatalytic plastic conversion, including enabling pre‐treatments, key reaction pathways, and the thermodynamic and kinetic challenges on depolymerization and product selectivity. Then, we summarize photocatalytic aromatic chemicals production from different plastics, including PET, PS, and other aromatic‐containing plastics and discuss how catalyst design enables high catalytic efficiency and product selectivity. Finally, the challenges and outlook for the development of photocatalytic plastic upcycling are presented, including the key factors governing high selectivity, improved photon utilization and reactor design, and the transition from pure plastic to real‐world plastic wastes. This review is expected to serve as a useful reference and provide guidance to accelerate the rational development of solar‐driven plastic upcycling.

## 2 Fundamental Mechanism of Photocatalytic Plastic Upcycling

Photocatalysis shows huge potential over other methods for plastic upcycling. Compared with pyrolysis, hydrogenolysis, and supercritical fluid treatment of plastics, which typically require high temperatures and/or high pressures, photocatalysis utilizes solar energy as the energy input and cleaves long polymer chains under mild conditions without additional carbon emissions [29]. Compared with solvolysis, enzymatic degradation, and biodegradation, which are generally applicable only to specific types of plastics, photocatalysis exhibits broad applicability and is less restricted by plastic structure [30]. In addition, solvolysis requires complex solvent systems, which may impose additional environmental burdens, whereas photocatalytic solvent systems are relatively simple and can be recycled. Compared with electrocatalysis, photocatalysis offers higher mass‐transfer efficiency, helping to overcome the kinetic limitations associated with long‐chain polymers [31]. Nevertheless, photocatalytic plastic upcycling still faces substantial challenges, and its performance remains far from satisfaction of practice application. These challenges include low solar‐to‐chemical energy conversion efficiency, instability of catalytic centers in solvents, and broad product distributions. Therefore, understanding the photocatalytic mechanism can provide important guidance for photocatalyst design and reaction‐process optimization.

**FIGURE 1:**
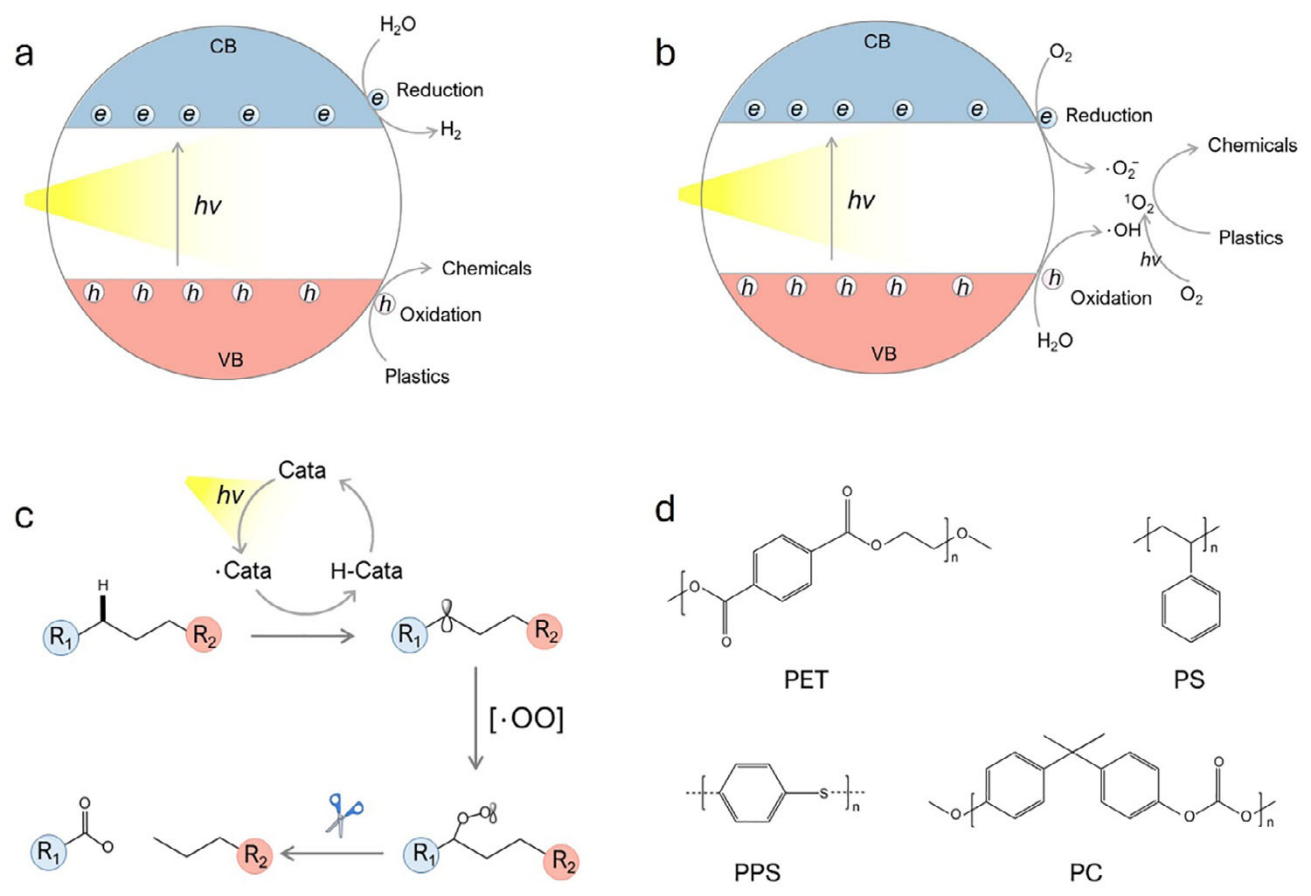
Schematic illustration of the (a,b) heterogeneous system and (c) homogeneous system. (d) Structure illustration of PET, PC, PS, and PPS.

Based on the catalytic system, photocatalytic plastic upcycling can proceed through heterogeneous or homogeneous catalysis. In heterogeneous systems, two principal reaction pathways are generally involved. One is an adsorption‐dominated pathway, which typically includes three steps: (i) photon absorption in the photocatalyst; (ii) photoexcitation to generate electron‐hole pairs; and (iii) direct oxidation of plastics by photogenerated holes (Figure 1a) [32]. The other is a radical‐mediated pathway, involving four key steps: (i) photon absorption by the photocatalyst; (ii) generation of photocarriers; (iii) formation of ROS; and (iv) subsequent reaction of the radicals with the plastics (Figure 1b) [1, 33]. Although the two pathways often coexist, the radical‐mediated process typically predominates in plastic conversion because the long‐chain nature of polymers leads to sluggish diffusion and limited adsorption on the photocatalyst surface. In homogeneous catalysis, the process is dominated by proton‐coupled electron transfer (PCET) (Figure 1c). Upon illumination, the photocatalyst is photoexcited and can directly abstract a hydrogen atom from C─H or O─H bonds in the polymer chain via hydrogen‐atom transfer, generating a radical intermediate, which is followed by a scission step, leading to chain fragmentation and subsequent conversion [34]. Homogeneous photocatalysis exhibits advantages in mass transfer. However, photocatalyst deactivation arising from photobleaching or radical‐induced side reactions compromise its stability [35]. In addition, radical intermediates undergo unrestricted diffusion, resulting in diminished selectivity and posing significant challenges in preserving aromatic structures while suppressing ring‐opening and over mineralization.

Different plastics that contain aromatic structures exhibit distinct depolymerization energetics and pathways due to their chemical structures (Figure 1d) [36]. PET and PC are condensation polymers, whose depolymerization mainly involves cleavage of C─O bond (∼ 443 kJ/mol) [37]. PPS is also a condensation polymer, and its depolymerization proceeds predominantly via C─S bond (∼ 306 kJ/mol) cleavage [38]. PS is an addition polymer, and its depolymerization requires scission of C─C bonds (∼ 347 kJ/mol) [39]. These robust chemical bonds require a high reaction potential to enable bond cleavage, imposing a thermodynamic barrier to polymer depolymerization. In addition, the polarity of plastics varies with their backbone chemistry, which influences their behavior in catalytic systems [40]. PS is non‐polar polymer due to its hydrocarbon backbone and phenyl substituents, leading to poor hydrophilicity in polar solvents. PPS is also hydrophobic polymer. Although the thioether linkage is intrinsically polarizable, it does not impart substantial polarity to the polymer backbone, thus PPS exhibits limited interfacial interactions with polar catalytic surfaces. In contrast, PET and PC contain ester or carbonate groups, respectively, introducing moderate polarity. These polar functionalities enhance solvent compatibility in polar solvents. Differences in polarity among plastics can provide guidance for the selection of the reaction medium. Moreover, the extended and entangled chain conformations of polymer molecules result in sluggish mobility in the reaction medium, giving rise to kinetic limitations in the catalytic process [41]. As a result, plastic depolymerization and oxidation can scarcely rely on simple molecular diffusion of the reactants to drive the reaction forward. Therefore, photocatalytic plastic upcycling is primarily governed by the diffusion of the catalyst and/or reactive species, rather than by the diffusion of polymer chains.

To enhance catalytic efficiency and mitigate the kinetic constraints imposed by long polymer chains, some studies employ pre‐treatment strategies to broken plastics into shorter polymer fragments prior to photocatalysis [24, 42]. These fragments are then subjected to subsequent photocatalytic depolymerization and oxidation. Common pre‐treatment strategies include solvolysis and thermal treatment. Solvolysis is primarily applicable to polyester‐based polymers, which can undergo alkaline hydrolysis in basic medium, leading to cleavage of C─O bonds [43]. For example, PET can be hydrolyzed in NaOH or alcoholic solutions, where OH^‒^ acts as a nucleophile and attacks the carbonyl carbon of the ester group to cleaved C─O bond, yielding terephthalate salts and ethylene glycol (EG). Thermal pre‐treatment is typically employed for polymers that are recalcitrant to hydrolysis or solvolysis, such as PS and PPS [44]. Mild thermal pre‐treatment can induce partial pyrolytic scission of polymer backbones, thereby reducing long‐chain polymers to lower‐molecular‐weight fragments. These pre‐treatment steps can substantially improve reaction kinetics and thereby enhance catalytic efficiency. However, they inevitably introduce additional energy input and processing steps, increasing the overall cost of plastic recycling. In addition, photocatalytic recovery of aromatic chemicals demands consideration not only of catalytic efficiency but also of product selectivity. During polymer depolymerization and oxidation, preserving benzene ring is essential to favor the formation of target aromatic products, while suppressing ring‐opening and mitigating over‐oxidation. The bond dissociation energies of C─C and C─H bonds at different positions in polymers directly influence the dehydrogenation and bond‐cleavage sites. For example, the bond dissociation energies (BDE) of the main‐chain C─C bonds in PS are ∼ 347 kJ/mol. In contrast, the BDE of the C─C_aromatic_ bonds are ∼ 415 kJ/mol [45]. The C─C bonds within the aromatic ring are more stable, thus β‐scission is more favorable in PS degradation. The benzylic C─H bond is easier to be activated due to the connection with phenyl ring, compared to the methylene C─H bonds and aromatic C─H bonds. Thus, hydrogen atom transfer (HAT) process is more likely to occur at benzylic C─H. However, reported studies indicated that insufficient oxygen species supply during the reaction can promote the formation of photoexcited triplet benzene species, leading to detachment of the phenyl ring rather than β‐scission, thereby suppressing the formation of BA [46]. In addition, the polarity of radical species can also affect the site selectivity of HAT process. For PS, electrophilic radicals, such as ∙Cl and some highly oxidizing radical species, tend to abstract H atom from electron‐rich benzylic C─H site. The resulting benzylic radicals can be stabilized by resonance with the phenyl ring, making this process both kinetically and thermodynamically favored. In contrast, nucleophilic radicals generally prefer to activate relatively electron‐deficient C─H bonds. Since PS does not contain strongly electron‐deficient C─H sites, nucleophilic radicals are less effective than electrophilic radicals in activating the C─H bond. For PET, electrophilic radicals preferentially abstract H atom from the C─H bonds in the EG segment, while nucleophilic radicals are more likely to interact with the electron‐deficient aromatic ring or carbonyl groups. Nevertheless, under strongly oxidative conditions, highly reactive and non‐selective radicals (such as ∙OH) may induce non‐selective activation and disrupt the integrity of the aromatic ring. To provide a clearer understanding of the kinetic barriers associated with bond cleavage in different polymers, the BDE of key bonds under various photocatalytic systems are summarized in Table 1.

**TABLE 1:** The BDE of key bonds under various photocatalytic systems.

Polymer	Bond	BDE	Radicals	References
PET	O─C(H_2_/∙O_2_^−^)	135.95 (kJ/mol)	∙O_2_^−^	[47]
	(H_2_/∙O_2_^−^)C─C(H_2_)	235.73 (kJ/mol)	∙O_2_^−^	
	(H_2_)C─O	200.81 (kJ/mol)	∙O_2_^−^	
PS	(Ph)C─H	10.8 (kcal/mol)	∙OH	[48]
	(Ph)C─H	14.7 (kcal/mol)	PhCO_2_∙	
	(Ph)C─H	26.3 (kcal/mol)	^1^O_2_	
	(Ph)C─H	29.3 (kcal/mol)	HO_2_∙	
	(Ph)C─H	34.7 (kcal/mol)	∙O_2_^−^	
PS	(Ph)C─H	20.3 (kcal/mol)	Catalyst radical (fluorenone)	[49]
	C─C (β‐scission)	3.9 (kcal/mol)	Catalyst radical (fluorenone)	
PS model (*trans*‐3)	(Ph)C─H	7.3 (kcal/mol)	Catalyst radical (AQ)	[50]
PS model (*cis*‐3)	(Ph)C─H	7.9 (kcal/mol)	Catalyst radical (AQ)	
PS	(Ph)C─H	2.22 V (SHE)	Catalyst radical (acridinium salts)	[51]
PS model (styrene dimer)	(Ph)C─H	4.8 (kcal/mol)	^1^O_2_	[52]
	(Ph)C─H	7.7 (kcal/mol)	∙OH	
	(Ph)C─H	35.8 (kcal/mol)	∙O_2_^−^	
	(Ph)C─H	40.2 (kcal/mol)	^3^O_2_	
	C─C (β‐scission)	4.0 (kcal/mol)	/	
PPS model (Cl‐fragment)	(Ph)Ca─S	12.8 (kcal/mol)	∙Cl	[53]
	(Ph)Cb─S	14.2 (kcal/mol)	∙Cl	
PSF model (Cl‐fragment)	(Ph)C─S(O_2_)	3.6 (kcal/mol)	∙Cl	[54]

## 3 Upcycling PET to Aromatic Chemicals

PET is an important polyester plastic, which is extensively used in packaging and textile fibers owing to its high optical transparency and mechanical toughness, accounting for ∼ 8% of global plastic production [42, 55]. Nevertheless, polyesters exhibit the highest rates of production, consumption, and disposal among major plastic classes, contributing to more than half of plastic waste streams, while the end‐of‐life recycling rate of PET remains only ∼ 25% [56]. Thus, the recovery of aromatic chemicals from waste PET offers benefits in mitigating plastic pollution and advancing a circular economy. PET is produced via the polycondensation of TPA and EG, which shows huge potential to recover TPA monomer or other aromatic chemicals.

**FIGURE 2:**
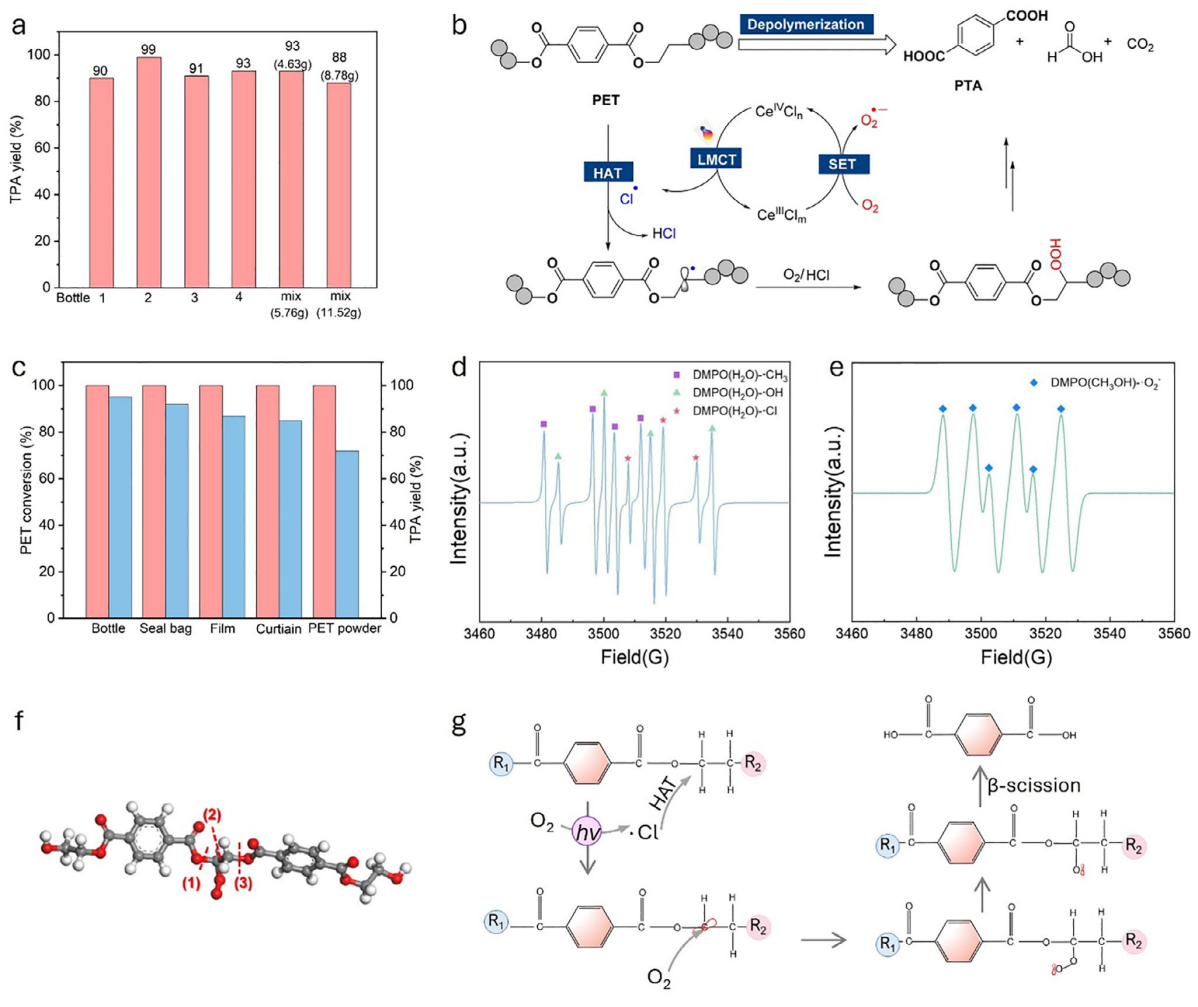
(a) TAP yield from PET waste from daily life. Reaction conditions (bottle 1–4): FeCl_3_ (0.05 mol%), NH_4_Cl (5 mol%), PET (96 mg, 0.5 mmol based on the single repeat unit), HFIP (2 mL, recycled), open air, LEDs (365 nm, 20 W), room temperature (rt), 24 h. Reaction conditions (mix): FeCl3 (0.05 mol%), NH4Cl (5 mol%), PET (1.82 g), HFIP (as noted, recycled), open air, room temperature (rt), 24 h, LEDs (365 nm, 20 W for 5.76 g; 400 nm, 50 W for 11.52 g). Reproduced with permission [57]. Copyright 2024, Wiley‐VCH. (b) Plausible mechanism. Reproduced with permission [58]. Copyright 2025, The Royal Society of Chemistry. (c) Conversion rates of PET plastic degradation over different types of photocatalysts. (d) EPR spectra of the product solution using deionized water as solvent. (e) EPR spectra of the product solution using methanol as solvent. (f) Calculated bond dissociation energies. (g) Proposed pathways of PET depolymerization. Reproduced with permission [47]. Copyright 2026, Elsevier.

There are generally two strategies for converting PET into aromatic chemicals: (1) direct conversion of polymers into high‐value chemicals; (2) depolymerization of polymers into monomers, oligomers, or other derivatives, followed by their recycling into high‐value chemicals in a closed‐loop system. Metal chlorides have demonstrated excellent performance in the direct depolymerization of PET plastics. Jiang et al. reported an FeCl_3_‐catalysed route for recovering the TPA monomer from PET, assisted by an additive (NH_4_Cl) and O_2_, using hexafluoroisopropanol (HFIP) as the solvent [57]. By optimizing the loadings of the catalyst and additive, the conversion of PET (Mw = 25 kg/mol) achieved 100% with 91% TPA yield. Then, the feedstock was extended from laboratory‐grade pure plastic to real‐world PET waste. Four PET sourced from commercial beverage bottles delivered conversions above 95% and TPA yields exceeding 90% (Figure 2a). Given the practical difficulty of sorting real‐world plastic waste, a mixed feedstock comprising the four PET wastes was further evaluated. In this case, 8.78 g of TPA was recovered from 11.52 g of waste plastic, demonstrating the feasibility of monomer recovery from realistic waste streams (Figure 2a). HFIP can be readily recovered and recycled as the reaction solvent via simple distillation, which can substantially reduce the environmental burden associated with solvent use. In addition, CeCl_3_ catalyst in the presence of HCl and O_2_ also enabled recovery of TPA from PET (Mw = 15 kDa), affording a TPA yield of 91% after 24 h [58]. Prolonging the reaction to 36 h resulted in only a marginal improvement, increasing the yield to 92%. The reaction system was subsequently evaluated at scale using post‐consumer eight PET plastic products and four PET fiber wastes. Despite the increased feedstock complexity, efficient depolymerization was maintained, delivering TPA yields over 78% after 24 h. These results underscore the robustness of the system and its tolerance towards the structural and compositional heterogeneity characteristic of real‐world PET waste streams. Mechanistic studies suggested that these catalytic systems are primarily driven by photoinduced oxidation of chloride to generate chlorine radicals (∙Cl). The ∙Cl species can abstract H atom from PET, producing HCl and concomitantly facilitating C─O bond scission along the polymer backbone. Subsequently, the in situ formed HCl can re‐coordinate to the metal center, regenerating the active catalysts and closing the catalytic cycle (Figure 2b). Owing to its high H‐abstraction propensity, ∙Cl effectively promotes key PCET steps to accelerate the reaction kinetics. However, metal center is prone to ligand exchange and competitive coordination by other ligands (e.g., solvent molecules and reaction‐derived oxygenates) under sustained illumination, which perturbs the active speciation and leads to catalyst deactivation. This deactivation is exemplified by the CeCl_3_ system, where the apparent efficiency increases by only ∼1% during the third 12 h reaction period. In addition, the strong oxidative reactivity of ∙Cl can render the process to over‐oxidation. The low‐carbon fragments formed alongside TPA during PET depolymerization are further oxidized to CO_2_, leading to additional CO_2_ emissions. To address the stability of catalyst associated with ∙Cl chemistry, He et al. reported a core‐shell Fe_3_O_4_@CeO_2_ heterojunction photocatalyst, which achieved 100% conversion of PET (plastic bottle feedstock) and a TPA yield of up to 95% in HFIP with HCl as the reaction medium after 48 h (Figure 2c) [47]. In addition, the seal‐bag, film, curtain, and powdered PET as feedstocks also delivered TPA yields of 92%, 87%, 85%, and 72%, respectively, which demonstrated the generality of the catalytic activity across diverse PET feedstocks. Mechanistic studies revealed that HCl, used as an additive, can generate the ∙Cl to promote C─H bond activation. Fe_3_O_4_@CeO_2_ can produce superoxide radicals (∙O_2_^−^), which assisted in C─O bond cleavage to accomplish PET depolymerization (Figure 2d,e). Specifically, Ce^4+^ was reduced to Ce^3+^ by photogenerated electrons, then Ce^3^^+^ acted with ∙O_2_^−^ to produce oxygen radicals (∙O) to cleave C─O bond in the polymer backbone. Ce^3+^ was then oxidized back to Ce^4+^ to complete Ce^4+^/Ce^3+^ redox cycle (Figure 2f,g). The synergistic involvement of ∙Cl and ∙O_2_^∙^^−^ promotes PET depolymerization to TPA, effectively mitigating catalyst deactivation. However, the highly oxidative ∙Cl still induce over oxidation of low‐carbon fragments, leading to the formation of low‐carbon gaseous products. Moreover, Fe_3_O_4_@CeO_2_ exhibited stability over 192 h, but the TPA yield still decreased by 10%. The ∙Cl can also induce poisoning of active sites in heterogeneous systems, leading to catalyst deactivation [59]. The decrease in TPA yield might be attributed to poisoning of a fraction of the catalytic active sites. The reported metal chlorides photocatalysts for PET upcycling mainly exhibit strong visible‐light responsiveness and can rapidly generate ∙Cl species upon photoexcitation, enabling high TPA yields within a short reaction time. However, the catalytic efficiency may decrease once the ∙Cl species are deactivated with prolonged reaction. Therefore, the development of ∙Cl‐dominated catalytic process requires catalysts with high structural stability and strong resistance to poisoning.

**FIGURE 3:**
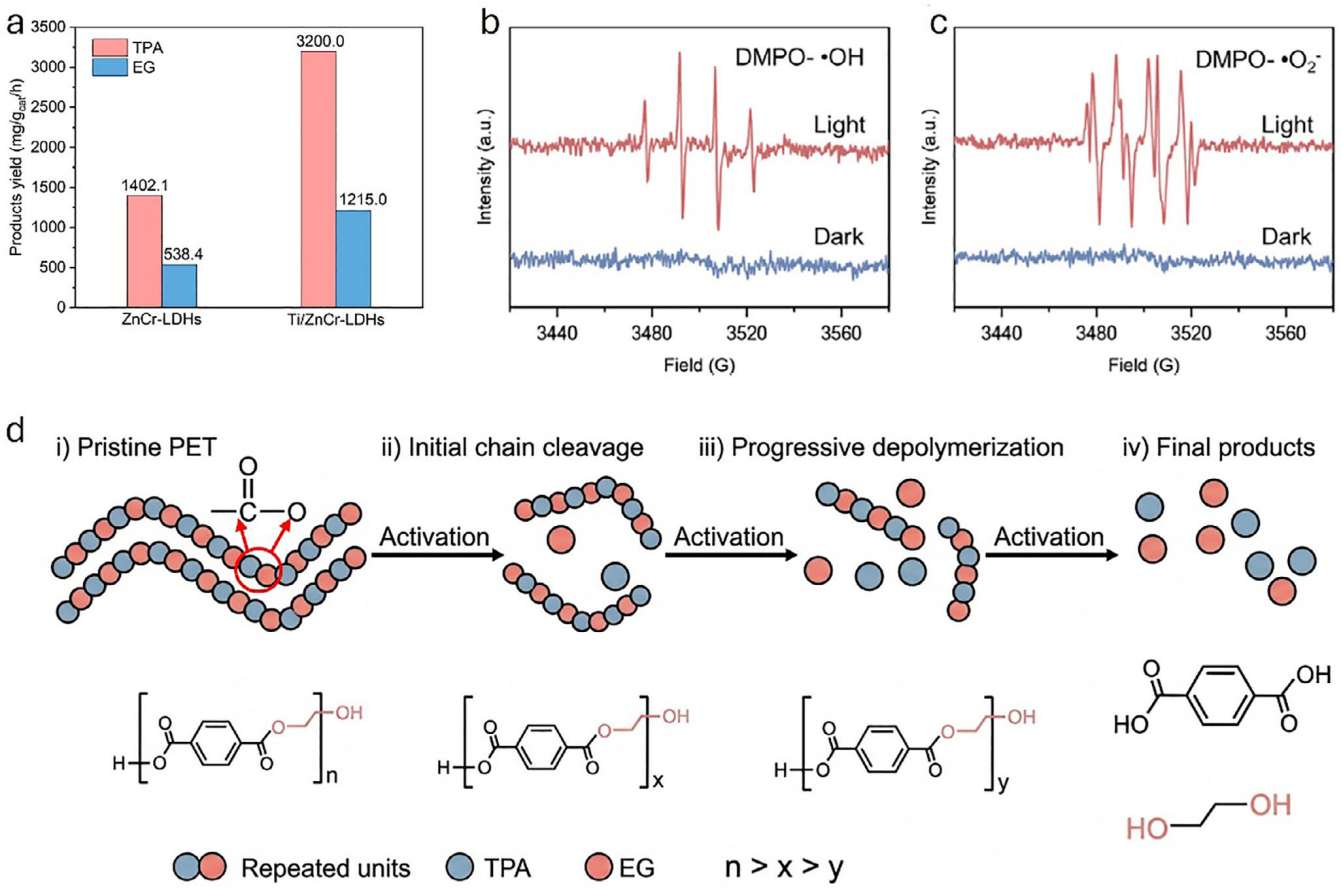
(a) TPA production and EG production over ZnCr‐LDHs and Ti/ZnCr‐LDHs under light irradiation. ESR spectra of (b) DMPO‐∙OH and (c) DMPO‐∙O_2_^−^ for Ti/ZnCr‐LDHs. (d) Proposed depolymerization pathway of PET. Reproduced with permission [60]. Copyright 2025, Wiley‐VCH.

In addition to ∙Cl that can drive PET depolymerization, ROS and certain photogenerated reactive intermediates also exhibit high catalytic activity. To overcome the over oxidation and catalysts poisoning of ∙Cl‐dominated catalytic processes, Wu et al. developed M/ZnCr‐LDHs (M = Ag, Cu, Mo, and Ti) photocatalysts to drive the PET depolymerize through robust ROS generation [60]. Among all M/ZnCr‐LDHs, Ti/ZnCr‐LDHs delivered the optimal catalytic performance, achieving 100% PET conversion in alkaline reaction medium (pH = 14). The yields of EG and TPA reached 1215 and 3200 mg/g_cat_/h, respectively, over 2 times higher than those of the pristine ZnCr‐LDHs (Figure 3a). These results indicated that introducing Ti plays a pivotal role in modulating the photocatalytic behavior, including light absorption, charge transport, and surface catalytic activity. Then, the substrate loading was increased from 100 to 300 mg, PET conversion remained at 100%. Further increasing the PET amount to 400 and 500 mg reduced the conversion to 83% and 69%, respectively, which was attributed to mass‐transfer limitations. Owing to the long‐chain structure nature, polymer molecules exhibit sluggish diffusion kinetics in solution. The reaction efficiency is governed not only by intrinsic catalytic activity but also by mass transport at higher substrate concentrations [61]. Mechanistic studies indicate that Ti doping effectively lowers the interfacial charge‐transfer resistance and prolongs the carrier lifetime, thereby facilitating photocatalytic redox reactions. Meanwhile, Ti/ZnCr‐LDHs can efficiently generate ∙OH and ∙O_2_^−^ species under illumination to drive PET depolymerization (Figure 3b,c). This process proceeds through four consecutive steps (Figure 3d): (i) the pristine PET polymer is first activated by ∙OH and ∙O_2_^−^; (ii) initial chain scission of the ester bonds occurs, producing oligomeric fragments; (iii) continued depolymerization further shortens the chain length; and (iv) complete conversion yields the monomeric end‐products, including TPA and EG. Compared with ∙Cl, ∙OH, and ∙O_2_^−^ exhibit a relatively milder oxidative capability, enabling better retention of the PET depolymerization products both TPA and EG. Therefore, development of ROS‐dominated catalytic processes represents a promising pathway for polymer monomer recovery. Furthermore, Zhang et al. reported an organic photocatalytic upcycling strategy using the phenothiazine derivative PTH‐3CN [48]. This system selectively deconstructs over 80% of plastics into high‐value small‐molecule products such as carboxylic acids under visible light and air without any metal or additives. In particular, post‐consumer PET bottles can be depolymerized in the presence of HFIP as solvent, enabling recovery of TPA with 93% isolated yield. The high catalytic efficiency originated from consecutive photoinduced electron transfer (conPET) process, in which PTH‐3CN served as pre‐catalyst and was transformed into the catalytically active triarylamine species. Upon two‐step photoexcitation, a highly oxidizing [PC^∙+^]^*^ was generated, in combination with oxygen species, which drive polymer depolymerization and enables TPA recovery. This two‐step electron‐transfer pathway and the highly reactive radical‐driven bond‐cleavage process provide a useful reference for the rational design of photocatalysts. Overall, LDHs and PTH‐3CN also show excellent performance and maintain their catalyst structures during long‐term reactions. However, the visible‐light responsiveness of these catalysts is primarily governed by their band structures. During charge‐carrier separation, electron‐hole recombination remains a major limitation. Thus, their visible‐light utilization is generally lower than that of metal chlorides. Their catalytic activity is strongly affected by mass transfer. Meanwhile, light‐shielding effects may occur and reduce the overall activity. Therefore, visible‐light utilization, mass‐transfer and dispersion are challenges for their scaling up.

As typical ester‐linked polymer, PET is predominantly depolymerized via C─O bond cleavage. Therefore, developing catalysts that favor site‐selective C─O activation represents one of the key directions for advancing PET upcycling. Beyond highly oxidative ∙Cl and ROS species, Lewis‐acid sites have been reported to exhibit excellent performance in promoting C─O bond polarization [62, 63]. Thus, designing catalysts with tailored Lewis‐acid/base sites is a promising strategy to enhance PET depolymerization. In addition, engineering hydrogen‐bonding networks, catalytic center microenvironments, and interfacial charge regulation can lower the activation barrier for C─O scission, enhance selectivity towards ester bonds, and suppress product loss arising from competing C─C cleavage and over‐oxidation to improve catalytic activity and selective [64, 65, 66]. Reaction engineering should also be integrated with the intrinsic properties of PET. Given mass‐transfer limitations arising from long‐chain and semi‐crystalline structure in PET, it is desirable to develop greener solvent systems, such as recyclable HFIP, and solid‐liquid interfacial activation strategies (e.g., surface hydroxylation) to improve conversion efficiency. Meanwhile, combining in situ/operando characterization, including in situ Fourier‐transform infrared spectroscopy (FTIR), electron paramagnetic resonance (EPR), and mass spectrometry to establish correlations among bond scission‐intermediates‐side reactions, will help identify the rate‐determining‐step and mechanism process to guide the development of energy‐efficient, impurity‐tolerant, and scalable processes for highly selective monomer recovery from PET.

## 4 Upcycling PS to Aromatic Chemicals

PS is an important styrenic plastic that is extensively used in packaging and building insulation owing to its light weight, good processability, and durability, accounting for ∼ 7.5% of total global plastic production [67]. Nevertheless, PS is also one of the most challenging commodity plastics to recycle due to high solvent resistance and non‐biodegradability, its recycling rate is only ∼ 6% [68]. PS is an addition polymer composed of C and H, where its polymer backbone consists of saturated C─C bonds with pendant phenyl rings, rendering PS essentially non‐polar. As a result, PS is insoluble in water and can only be dissolved or swollen by several organic solvents, such as aromatic hydrocarbons, halogenated hydrocarbons, and ketones. However, the phenyl moiety accounts for ∼ 74 wt.% of PS, providing huge potential for the recovery of aromatic chemicals from PS waste.

**FIGURE 4:**
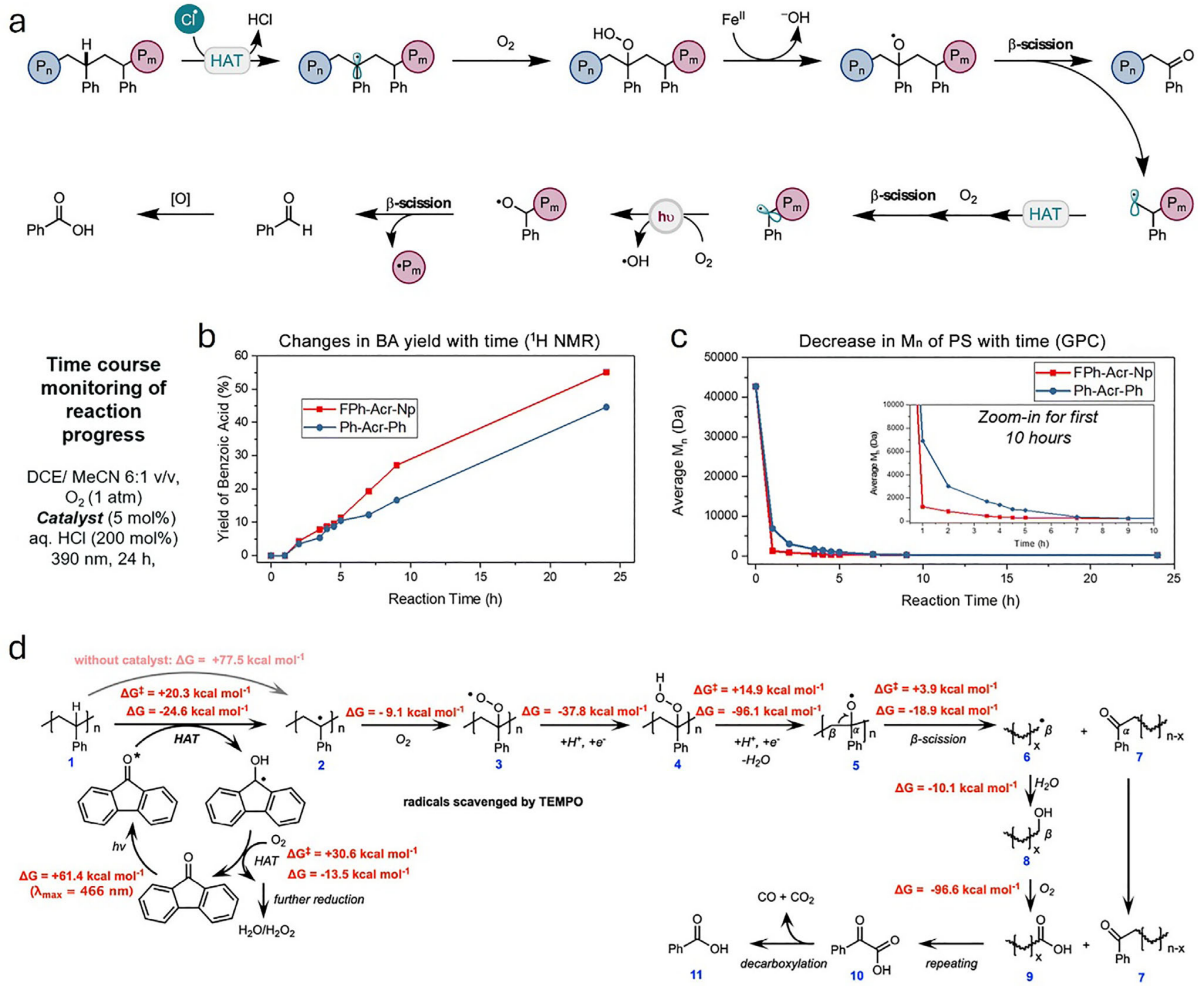
(a) Proposed mechanism for both chain cleavage and major pathways for benzoyl product generation. Reproduced with permission [70]. Copyright 2022, American Chemical Society. Time course experiments showing the evolution of (b) PS Mw and (c) BA yields with increasing reaction duration. Yields are calculated from an average of two repeat experiments (*yields in parentheses consider removal of insoluble additives such as dyes). Reproduced with permission [51]. Copyright 2024, The Royal Society of Chemistry. (d) Proposed reaction mechanism. Free energy changes (ΔG) and kinetic energy barrier (ΔG^‡^) were calculated by DFT with a styrene dimer model compound. Reproduced with permission [49]. American Chemical Society.

Photocatalytic upcycling of PS mainly includes depolymerization and oxidation processes, which occur simultaneously and proceed via C─H bond oxidation and C─C bond scission, with the major oxidation products including BA, benzene, and phenol [28, 69]. Current mechanistic studies suggest that depolymerization and oxidation of PS rely primarily on radical‐driven HAT, where radicals abstract H atom from the polymer backbone, after which oxygen species deliver O atoms to form C─O bonds, promoting C─C bond oxidation and scission. The key radical species responsible for the excellent catalytic activity include ∙Cl, catalyst‐derived radicals (∙Cata), ∙O_2_^−^, and ^1^O_2_. For example, Oh et al. reported FeCl_3_ catalyzed strategy to convert PS (> 90 kg/mol) into 23 mol% benzoyl products [70]. The product distribution indicates that BA, benzaldehyde, benzoyl chloride, and acetophenone are the major products, whereas benzene and chlorobenzene are detected only in trace amounts. The detection of chlorobenzene suggests that a fraction of ∙Cl is quenched during the reaction and becomes incorporated into products. To clarify the key role of ∙Cl, benzophenone was used in place of FeCl_3_, the yield of aromatic products decreased sharply, indicating that ∙Cl is highly active species for PS conversion, although their limited stability constrains overall performance. Kinetic studies showed that, at the initial stage of the reaction, the amount of benzaldehyde increased within the first 12 h and then gradually decreased over longer reaction times, whereas the amount of BA increased continuously with time and ultimately became the dominant product. Control experiments were performed in the presence of 10 wt.% 4‐*tert*‐butylbenzaldehyde to confirm whether benzaldehyde was converted into BA. The results revealed that benzaldehyde was progressively oxidized to the corresponding BA or benzoyl chloride. Notably, the benzoyl chloride began to decline after 12 h, which may be attributed to hydrolysis to BA with regeneration of chloride ion. Thus, the catalytic pathway was proposed, FeCl_3_ generates ∙Cl upon photoexcitation that abstract H atoms from the Cα, then C─H bond is oxidized to C─O bond in the presence of O_2_, followed by β‐scission to yield aromatic oxygenates (Figure 4a). However, the inherent instability of ∙Cl limits their applicability in long‐term catalysis. To extend the lifetime and improve the regeneration of ∙Cl, acridinium salts catalysts (Ph‐Acr‐Ph, Ph‐Acr‐Me, and Mes‐Acr‐Me) were proposed to work synergistically with ∙Cl for PS depolymerization [51]. Upon photoexcitation, acridinium salts generated the excited‐state radical Acr^+^*, which promoted the ∙Cl/Cl^−^ redox cycle and thereby accelerated the HAT process. With this synergistic effect, the average molecular weight (Mw) of PS dropped sharply within the first hour (Figure 4b), and PS was ultimately converted into BA and formic acid. Notably, BA formation was negligible during the first hour but increased progressively with extended reaction time (Figure 4c), suggesting that PS was initially cleaved into smaller fragments that were subsequently upgraded to BA. In addition, the synergy of acridinium salts and HCl in PS depolymerization enabled a reduced catalyst loading (< 5 mol%), which maintained high activity (up to 55%) while mitigated the instability associated with ∙Cl, thereby improving practical applicability. Besides ∙Cl, organic photocatalysts containing C═O bonds are also reported excellent catalytic activity for PS depolymerization. The organic photocatalysts can generate photoexcited C═O* radicals under light irradiation to exhibit strong H‐abstraction capability to form C─OH for C─H bond oxidation. For example, Li et al. reported the fluorenone as an organic photocatalyst for oxidative depolymerization of PS, where the Mw of PS decreased from 260 000 to ∼ 100–200 within 16 h [49]. Product distribution showed that BA was formed in a yield of ∼30%, together with ethyl benzoate (∼ 10%), acetophenone (∼ 6%), and benzaldehyde (∼ 1%). Gaseous products were also detected, including CO (21%) and CO_2_ (7.5%). Notably, most of the fluorenone (∼ 95%) remained stable structure after 48 h, and it could be recovered from the 24 h reaction mixture with an isolated yield of 95%. The recovered photocatalyst still afforded BA in 34% yield in a second 24 h cycle, exhibiting the good photocatalytic activity and recyclability of fluorenone. Subsequently, gram‐scale reactions were carried out using a real‐world PS foam feedstock (Mw ≈ 250 000, PDI ≈ 4.2). Within 72 h, an overall aromatic product yield of 60 ± 4% (including 36 ± 2% BA) was obtained, demonstrating the practicality and scalability of this photocatalytic depolymerization strategy for recovering aromatics from PS waste. Mechanistic studies indicated that the ketyl radical generated from fluorenone effectively reduced both the free energy barrier of the HAT step (ΔG = −24.6 kcal/mol) and the kinetic barrier (ΔG^‡^ = + 20.3 kcal/mol) (Figure 4d). By contrast, the HAT step was highly endergonic (ΔG = + 77.5 kcal/mol) in the absence of the photocatalyst. The favorable thermodynamics and kinetics accelerated the subsequent β‐scission process of PS, corroborating the catalytic role of fluorenone. Nikitas et al. investigated the conversion of PS using a range of organic photocatalysts, finding that aromatic ketones afforded only 1.9‐6.0% BA, whereas anthraquinone (AQ) delivered an 18.8% isolated yield of BA [71]. After optimizing the reaction conditions, 28.2% yield of BA was obtained using 5 mol% AQ under 390 nm LED irradiation in air for 48 h. Using commercial PS as the substrate, a plastic spoon feedstock gave a BA yield of up to 51.4%. However, chlorinated polystyrene exhibited diminished performance with 13.1% yield of BA, which might be due to chlorine‐induced deactivation of the photocatalyst. Mechanistic studies suggested that the C═O bond in AQ can be photoexcited to form C═O* for abstracting the Cα‐H of PS to generate C─OH intermediate to oxidize the Cα─H bond. Finally, the C─OH intermediate was re‐oxidized by O_2_ back to C═O, completing the catalytic cycle. Moreover, an AQ derivative, sodium 1‐anthraquinonesulfonate (AQS), was also found to exhibit efficient PS depolymerization [50]. The AQS enabled a Hock‐type rearrangement of PS in the presence of a strong acid, directly converting PS into a phenolic derivative, phenyl acetate, while effectively suppressing BA formation. To the best of current knowledge, this work represents the first report of converting PS into phenolic derivatives. When using real‐world PS waste, phenyl acetate was obtained in yields of up to 17.2%, and no BA was detected. The use of acridinium salt catalysts in synergy with ∙Cl species can achieve excellent catalytic activity while improving catalyst stability. In addition, fluorenone‐based catalysts have also shown excellent recyclability. The development of these catalysts could provide valuable guidance for the scale‐up of photocatalytic PS upcycling.

**FIGURE 5:**
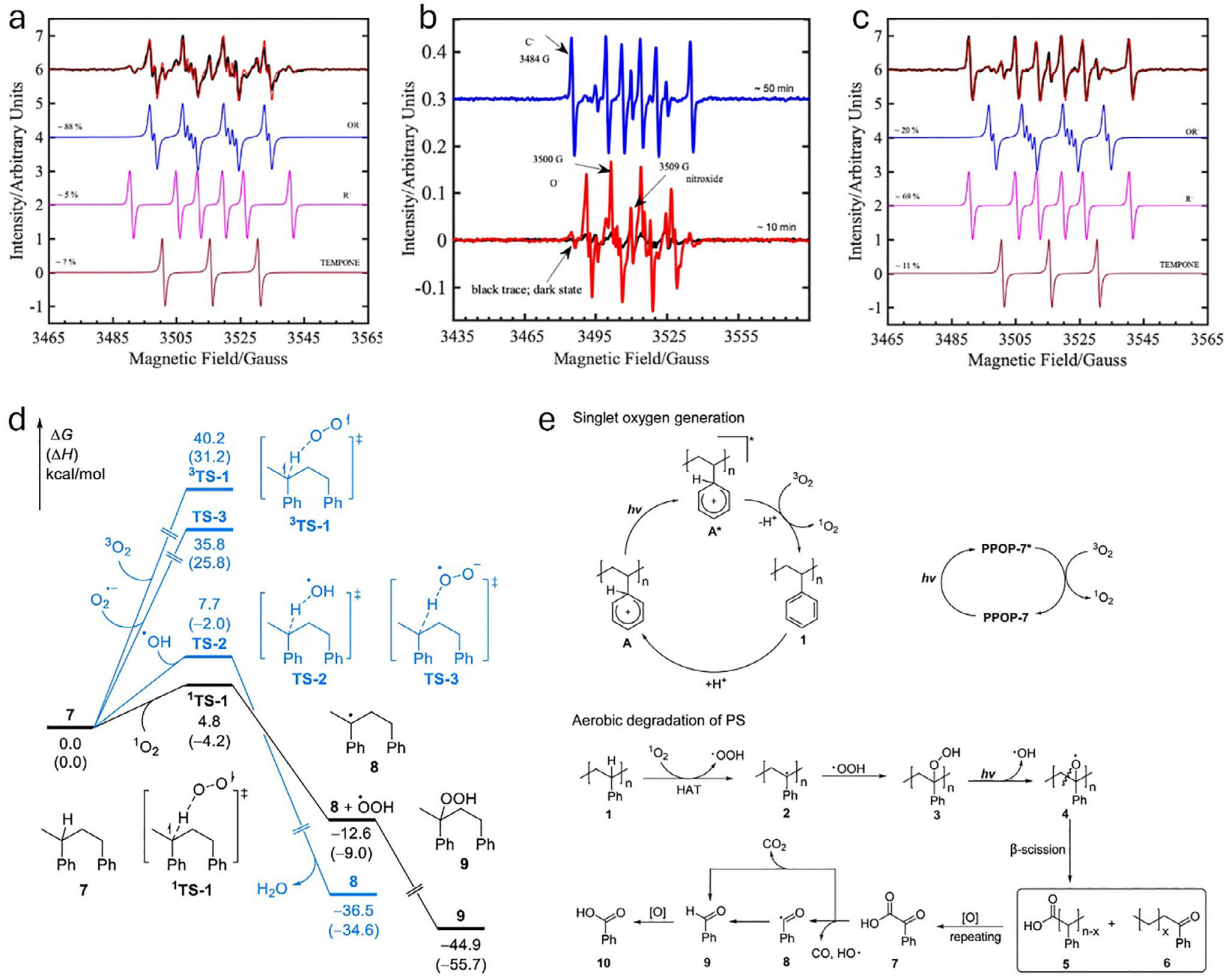
(a–c) cw EPR spectra from in situ irradiation at 405 nm (1 mW LED) of PS and a pTsOH∙H_2_O solution with 4‐oxo‐TMP and DMPO spin traps (1:4). Center: experimental spectra measured after 10 min (red) and 50 min (blue) irradiation. Left and right: simulated spectra of three separate components of a nitroxyl [g = 2.0056, aiso(14N) = 1. 5 mT], a carbon‐centered DMPO adduct [g = 2.0055, aiso(14N) = 1.45 mT, aiso(β‐1H) = 2.11 mT], and an oxygen‐centered DMPO adduct [g = 2.0057, aiso(14N) = 1.29 mT, aiso(β‐1H) = 1.03 mT, aiso(γ‐1H) = 0.13 mT]. Simulations (red) of the experimental spectra (black) after 10 and 50 min (left and right, respectively) are weighted sums of these three components. (d) Computational study of the HAT reaction of 1,3‐diphenylbutane 7 with various oxygen species. Reproduced with permission [52]. American Chemical Society. (e) Proposed reaction mechanism. Reproduced with permission [27]. The Royal Society of Chemistry.

ROS also exhibit excellent activity for C‐H oxidation in PS, especially ^1^O_2_ and superoxide ∙O_2_^−^. In contrast to ∙Cl and catalyst‐derived organic radicals, ROS are generated from O_2_ and/or H_2_O, which can be produced continuously during the reaction [72]. Their consumption or quenching does not compromise the stability of the photocatalyst. Thus, ROS‐driven pathways hold considerable promise for photocatalytic upcycling of PS. Huang et al. reported a low‐cost, acid‐driven strategy for PS depolymerization, where triflic acid, p‐Toluenesulfonic acid monohydrate (pTsOH∙H_2_O, 5 mol%), exhibited the selective aerobic depolymerization of PS (Mw = 192 000) using O_2_ (1 bar) as the oxidant under violet‐blue light (405 nm) irradiation, affording isolable products including formic acid (72%), BA (40%), and benzophenone (2%) [52]. Several acid catalysts were also evaluated, showing that methanesulfonic acid (CH_3_SO_3_H) and sulfuric acid (H_2_SO_4_) also promoted formation of the corresponding products, whereas the Lewis acids, Sc(OTf)_3_ and La(OTf)_3_, can also produce aromatic chemicals but with reduced yields. Using pTsOH·H_2_O as catalysts, commercial PS waste has been carried for further aerobic depolymerization. Ten real‐world PS items (e.g., cups, containers, and packaging) were rapidly cleaved by O_2_ to give aromatic products with high selectivity, delivering BA in up to 46% yield with an isolated yield of 45%. Moreover, when poly(4‐tert‐butylstyrene) (Mw = 50 000–100 000) was employed as the feedstock, 4‐tert‐butylbenzoic acid was obtained in 46% yield with a 50% isolated yield. Then radical‐trapping experiments were conducted to elucidate the reaction pathway, which indicated that ^1^O_2_ is the dominant ROS and plays a crucial role in PS depolymerization. DMPO trapping further suggested that O‐centred radicals form initially, followed by the evolution of C‐centred radicals, with the formation of these radicals being strongly promoted by both the PS substrate and the pTsOH∙H_2_O catalyst (Figure 5a–c). UV‐vis spectroscopy showed that neither PS nor pTsOH∙H_2_O alone exhibited an absorption band near 405 nm. However, a pronounced band appears at ∼ 408 nm upon mixing of PS nor pTsOH∙H_2_O, implying that a [PS—H^+^] adduct formed via PS‐pTsOH∙H_2_O interaction may act as a photosensitizer to trigger ^1^O_2_ generation under illumination. DFT calculations indicated that ^1^O_2_‐mediated HAT from the substrate C─H bond showed the lowest activation barrier, making ^1^O_2_ the most plausible initiating oxidant for PS depolymerization (Figure 5d). Following H abstraction, a stabilized benzylic radical is formed, which irreversibly combines with ∙OOH to generate a peroxide intermediate, thereby facilitating subsequent C─C bond scission. This work suggests that acid catalysts can activate O_2_ to ^1^O_2_through specific interactions with PS, unveiling an unconventional catalytic mechanism that offers guidance for future catalyst design. Man et al. proposed a synergistic system combining mesityl acridinium perchlorate (Mes‐Acr^+^ClO_4_^−^) as a photosensitizer with pTsOH∙H_2_O [73]. Under the optimized conditions, commercial PS (Mw ≈ 350 000 g/mol) was employed as the feedstock, delivering 73% yield of BA within 24 h under 365 nm LED irradiation. Then, commercial PS samples and real‐world PS waste was evaluated. A total of 15 styrenic plastics were converted to BA in 53–71% yield. Notably, the more chemically recalcitrant styrene‐acrylonitrile (SAN) copolymer was also readily upgraded to BA in 54% yield. Importantly, even mixed PS‐plastic feedstocks could be successfully transformed into BA (58%), highlighting excellent practicality and circumventing the need for plastic sorting. Radical‐trapping experiments indicated that ^1^O_2_ remains the dominant ROS in this work. Distinct from the acid‐only system, Mes‐Acr^+^ClO_4_^−^ is photoexcited to Mes‐Acr^+^ClO_4_^−^*, which transfers energy to ground‐state molecular oxygen (^3^O_2_) to generate ^1^O_2_. Porphyrin‐based porous organic polymers (PPOPs) served as photosensitizers with p‐TsOH·H_2_O as an additive can also promote the conversion of ^3^O_2_ to ^1^O_2_ to facilitate benzylic C‐H oxidation and subsequent C‐C bond scission [27]. Using PS (Mw ≈ 350 000 g/mol) as the feedstock, BA was obtained in 49% yield after 16 h, increasing to 62% after 48 h. Mechanistic studies revealed a cooperative catalytic role between the photosensitizer and pTsOH∙H_2_O (Figure 5e). Under acid assistance and photoexcitation, ^1^O_2_ is generated via energy transfer between O_2_ and the excited state of the photocatalyst. Then the ^1^O_2_ initiated radical oxidation through benzylic C‐H HAT, forming a peroxide intermediate that subsequently undergoes O─O bond cleavage and β‐scission, progressively cleaving the polymer chains. Through intermediates such as benzoylformic acid and benzaldehyde, the process ultimately affords BA. In addition, Y‐type zeolite with H^+^ as cation also exhibited ^1^O_2_‐dominated catalytic activity for PS depolymerization [74]. BA yield of 30.6% was achieved within 15 h using commercial PS (Mw = 274 000) as the feedstock, and the BA yield remained stable over three consecutive cycles. Moreover, four commercial PS products all showed excellent reactivity, affording BA in yields of 25.1‐29.8%. As the dominant ROS, ^1^O_2_ has demonstrated excellent performance for PS upcycling across a range of catalytic systems.

**FIGURE 6:**
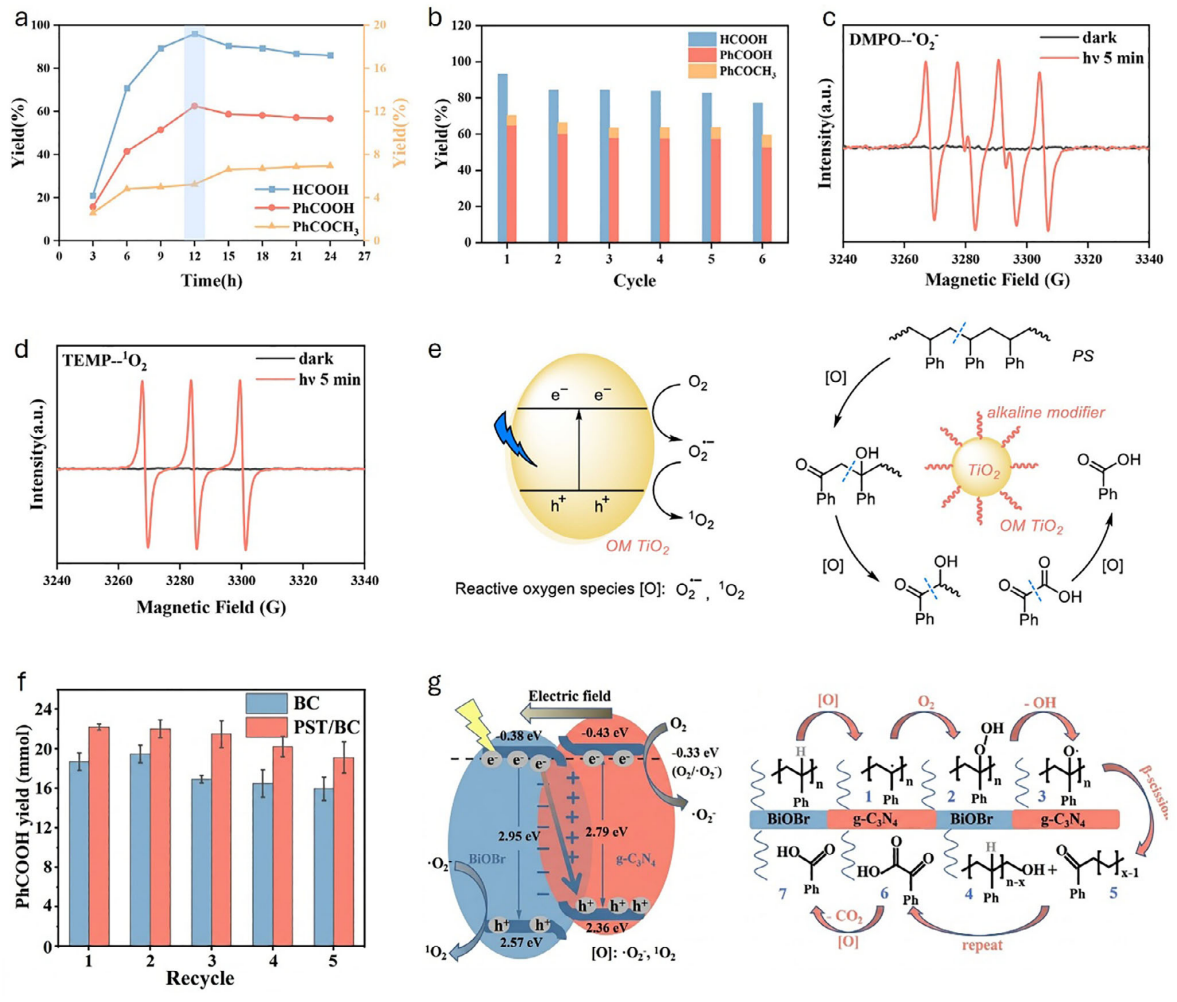
(a) The time curve of V‐POPs catalyzing the oxidation of PS. (b) Cycle test of V‐POPs. EPR detection of (c) ∙O_2_^−^ radicals formed in situ using DMPO and (d) ^1^O_2_ formed in situ using TEMP as a spin‐trapping agent with V‐POPs (10 mg) in DMF (1 mL) under irradiation. Reproduced with permission [77]. Wiley‐VCH. (e) Proposed mechanism for photocatalytic aerobic oxidation of PS on organic‐modified TiO_2_. Reproduced with permission [79]. American Chemical Society. (f) BC and PST/BC‐2 photocatalysis of PS over five cycles. (g) Charge transfer mechanism of PST/BC‐2 surface‐modified materials and possible photocatalytic conversion mechanism of PS. Reproduced with permission [80]. Elsevier.

∙O_2_^−^ have also demonstrated outstanding ability to promote C‐H activation and β‐scission of PS [75, 76]. PS upcycling processes synergistically driven by ∙O_2_^−^ and ^1^O_2_ exhibit high catalytic activity and stability relative to those driven by ∙Cl or catalyst‐derived radicals. Wang et al. synthesized 9‐fluorenone‐based porous organic polymer photocatalysts (V‐POPs), which catalyzed the aerobic oxidative degradation of PS under blue‐light irradiation (455 nm) at ambient temperature and pressure [77]. After 12 h of reaction, PS conversion over V‐POPs afforded formic acid (96%), BA (67%), and acetophenone (4%), with the BA yield being ∼ 3 times that obtained with 9‐fluorenone alone (Figure 6a). Moreover, V‐POPs exhibited excellent recyclability, maintaining high catalytic activity over 6 cycles without significant performance loss (Figure 6b). The catalytic system was subsequently extended to the oxidative upcycling of various industrial and post‐consumer PS wastes, delivering relatively high and stable BA yields (45%–63%). Photoelectrochemical properties of the photocatalysts were then investigated. Compared with conventional traditional porous organic polymer photocatalysts (F‐POPs), V‐POPs showed charge separation and transport capability. In addition, the valence band (VB) position of V‐POPs was determined to be 2.36 V (vs normal hydrogen electrode, NHE), higher than that of F‐POPs (2.16 V vs NHE), thereby providing a stronger thermodynamic driving force for oxidation reactions. Overall, the incorporation of rigid V‐POSs nanocages into the 9‐fluorenone‐based polymer framework simultaneously enhanced charge separation, promoted charge‐carrier transport, suppressed charge recombination, and optimized the band structure. These synergistic improvements collectively account for the outstanding photocatalytic performance of V‐POPs. Mechanistic investigations revealed that V‐POPs could activate oxygen (O_2_) to generate reactive oxygen species (∙O_2_^−^ and ^1^O_2_) (Figure 6c,d), which dominated the overall oxidation process and cleaved the PS main chain via a radical pathway to form oxygenated intermediates, ultimately converting them into organic acid products. A high‐surface‐area g‐C_3_N_4_ nanosphere catalyst prepared via flow‐assisted exfoliation and O‐doping (F‐g‐C_3_N_4_) has also been reported for PS upcycling [78]. Compared with pristine g‐C_3_N_4_, F‐g‐C_3_N_4_ exhibited markedly enhanced light absorption, charge separation capability, and charge‐carrier separation efficiency. In addition, both the short‐lived component (τ_1_) and long‐lived component (τ_2_) of F‐ g‐C_3_N_4_ decreased relative to those of pristine g‐C_3_N_4_, with τ_1_ decreasing from 0.8 ns to 0.5 ns and τ_2_ from 4.0 ns to 3.0 ns. Accordingly, the average lifetime decreased from 3.0 ns for g‐C_3_N_4_ to 2.4 ns for F‐g‐C_3_N_4_. Meanwhile, the contribution ratio of the short‐lived component decreased from 31% to 22%, whereas that of the long‐lived component increased from 69% to 78%. The short‐lived component is generally associated with the radiative decay of the excited state back to the ground state. Its reduced contribution indicates a decrease in the number of rapidly recombining charge carriers. The shortened average lifetime, together with the change in the contribution ratios of the lifetime components, collectively suggests that more non‐radiative transfer processes occurred for photogenerated charge carriers in F‐g‐C_3_N_4_. This may imply that its nanosphere structure shortened the carrier transport distance. Photoelectrochemical results also demonstrated that electron‐hole separation in F‐ g‐C_3_N_4_ was more efficient than in pristine g‐C_3_N_4_. Therefore, under the optimal catalytic conditions, F‐g‐C_3_N_4_ enabled the conversion of PS into acetophenone (AP) and BA, achieving a PS conversion of 63.2% within 8 h, with selectivities of 86.2% for AP and 13.8% for BA. Moreover, the catalyst maintained good photocatalytic activity over five consecutive cycles without obvious deactivation or structural change. Mechanistic studies revealed the photocatalytic conversion pathway of PS. PS molecules were first adsorbed onto the catalyst surface, followed by the generation of electron‐hole (e^−^‐h^+^) pairs under light irradiation. Conduction‐band electrons reacted with oxygen molecules to generate ∙O_2_^−^ (O_2_ + e^−^ → ∙O_2_^−^), while photogenerated h^+^ attacked the C‐H bonds of PS, activating them to form PS radicals and protons (PS + h^+^ → ∙PS + H^+^). Subsequently, protons reacted with ∙O_2_^−^ to generate HOO∙ radicals (H^+^ + ∙O_2_^−^ → HOO∙). Two HOO∙ radicals then coupled to form the H_2_O_2_ intermediate (2HOO∙ → H_2_O_2_). Under light irradiation, H_2_O_2_ further decomposed into ∙OH radicals (H_2_O_2_ → ∙OH + OH^−^). The ∙OH radicals then reacted with PS radicals to generate the intermediate PS–OH (∙PS + ∙OH → PS‐OH), which subsequently underwent O─H bond activation by valence‐band holes to form PS‐O∙ and H^+^ (PS‐OH + h^+^ → PS‐O∙ + H^+^). ∙O_2_^−^ and protons then reacted again to generate HOO∙ radicals. Thereafter, with the participation of ∙H radicals, the intermediate underwent C─C bond homolysis to produce AP, which was further oxidized to a small amount of BA. In this way, photocatalytic C─H activation and oxidative bond cleavage of polystyrene were achieved under mild conditions, selectively yielding acetophenone and BA. Peng et al. reported the use of a conventional metal‐oxide photocatalyst, TiO_2_ particles, for PS upcycling [79]. Surface modification of TiO_2_ with potassium stearate (PSA) and N,N‐diethyl‐3‐(trimethoxysilyl)propan‐1‐amine (DTSPA), affording PSA‐TiO_2_ and DTSPA‐TiO_2_, tuned the interfacial interaction between the highly polar TiO_2_ surface and the weakly polar hydrocarbon polymer, thereby promoting C─H oxidation and C─C bond cleavage of PS. Photocatalytic experiments subsequently showed that 97% of PS was converted into low‐molecular‐weight products within 4 h. Specifically, the weight‐average Mw decreased from 250 695 to 409/125, while the number‐average Mw decreased from 103 428 to 365/117, indicating that PS was efficiently degraded into oligomers and monomers. 5 types of PS waste could all undergo oxidation over PSA‐TiO_2_, affording 18–40.4 mol% of BA. Compared with PSA‐TiO_2_, DTSPA‐TiO_2_ exhibited recycling performance, delivering BA yields of 34.3–44.2 mol%. Mechanistic studies indicated that ∙O_2_^−^ and ^1^O_2_ were the main ROS. Compared with unmodified TiO_2_, both PSA‐TiO_2_ and DTSPA‐TiO_2_ exhibited stronger ∙DMPO‐O_2_^−^ signals, suggesting that these surface modifiers promoted O_2_ activation on the catalyst. Based on the above experimental results, a possible mechanism for photocatalytic PS oxidation was proposed. Upon light irradiation, e^−^‐h^+^ pairs were generated, after which ground‐state oxygen was first reduced by electrons to form ∙O_2_^−^ (Figure 6e). These ∙O_2_^−^ could either directly participate in the reaction as ROS or be further converted into ^1^O_2_ under h^+^‐mediated oxidation. The oxidation of PS was initiated by partial oxidation of alkyl C─H bonds and cleavage of C─C bonds, a process that was significantly promoted by the presence of basic surface modifiers on TiO_2_. Furthermore, polymers bearing carbonyl and hydroxyl functionalities could undergo continued oxidation to form intermediates analogous to 2‐hydroxyacetophenone and phenylglyoxylic acid, which were then rapidly oxidized on the TiO_2_ surface to BA. To further improve the separation efficiency of photogenerated e^−^‐h^+^ pairs in photocatalysts for PS upcycling, Wang et al. proposed the construction of surface‐modified BiOBr/g‐C_3_N_4_ (BC) heterojunction photocatalysts with potassium stearate (PST) [80]. Using PST/BC photocatalysts, a BA yield of 32.63% was achieved within 16 h, with no obvious decline in catalytic performance over 5 consecutive cycles (Figure 6f). The introduction of PST significantly altered the microstructure and surface properties of the BC composite. This PST‐modified BC photocatalysts enhanced the separation and transport of photogenerated e^−^‐h^+^ pairs by strengthening interfacial interactions. Meanwhile, the alkyl chains on the catalyst surface could effectively enrich PS molecules and their degradation intermediates, thereby markedly improving the selectivity towards BA formation. Using the optimized catalyst, the conversion of two commercial plastic products was also successfully achieved, affording BA yields of 31.65% and 29.59%, respectively. Mechanistic studies indicated that a built‐in electric field (BEF) directed towards BiOBr was formed at the heterojunction interface. This BEF caused upward band bending in g‐C_3_N_4_ and downward band bending in BiOBr. Under the action of this internal electric field, photogenerated electrons were more inclined to migrate from BiOBr to g‐C_3_N_4_. In addition, PST‐modified BC exhibited strong interfacial interactions, which further enhanced interfacial charge‐transfer capability and thereby promoted the separation and transport of photogenerated electrons. Because the conduction‐band potential of g‐C_3_N_4_ (−0.43 eV) is more negative than the reduction potential of O_2_/∙O_2_^−^ (−0.33 V vs NHE), conduction‐band electrons can reduce O_2_ to ∙O_2_^−^, while holes in the valence band of BiOBr can further oxidize ∙O_2_^−^ to generate ^1^O_2_. These photogenerated ROS can directly participate in the oxidation of PS, enabling C─H oxidation and C─C bond cleavage. A possible photocatalytic oxidation mechanism was subsequently proposed (Figure 6g). First, the alkyl chains on the surface of PST/BC could effectively enrich PS molecules and their degradation intermediates. In the presence of reactive oxygen species, PS underwent HAT to generate intermediate 1 containing benzylic radicals (R∙). This intermediate was then oxidized by O_2_ to form intermediate 2, which underwent dehydroxylation to yield intermediate 3 containing alkoxy radicals (RO∙). These relatively stable benzylic and oxygen‐centerd radicals induced β‐scission, leading to cleavage of the C─C bonds in the PS main chain and generating intermediates 4 and 5. These intermediates were further oxidized to form intermediate 6 or were directly converted into BA. In addition, intermediate 6 and other possible intermediates could also be further oxidized by oxygen or ROS to produce BA.

The recovery of aromatic chemicals from PS mainly relies on ∙Cl, carbonyl‐based organic photocatalysts, and ROS to drive the catalytic process. The dissociation efficiency of PS generally exceeds 90%, while the recovery yield of aromatic products ranges from 13% to 87%, with BA and AP being the major aromatic products. In general, ROS‐driven catalytic process exhibits higher catalyst stability than ∙Cl ‐driven and carbonyl‐based organic photocatalytic systems. Therefore, the design of photocatalysts that enable ROS‐driven catalytic recycling of PS represents one of the important future research directions.

## 5 Upcycling Other Plastic to Aromatic Chemicals

In addition to PET and PS, the two major classes of plastics containing aromatic structures, several other plastics with aromatic structures have also been considered for the photocatalytic recovery of aromatic chemicals. For example, polysulfones (PSFs), polyphenylene sulfide (PPS), and BPA‐derived polymers also possess aromatic structures, enabling the recovery of aromatic chemicals from these plastics.

**FIGURE 7:**
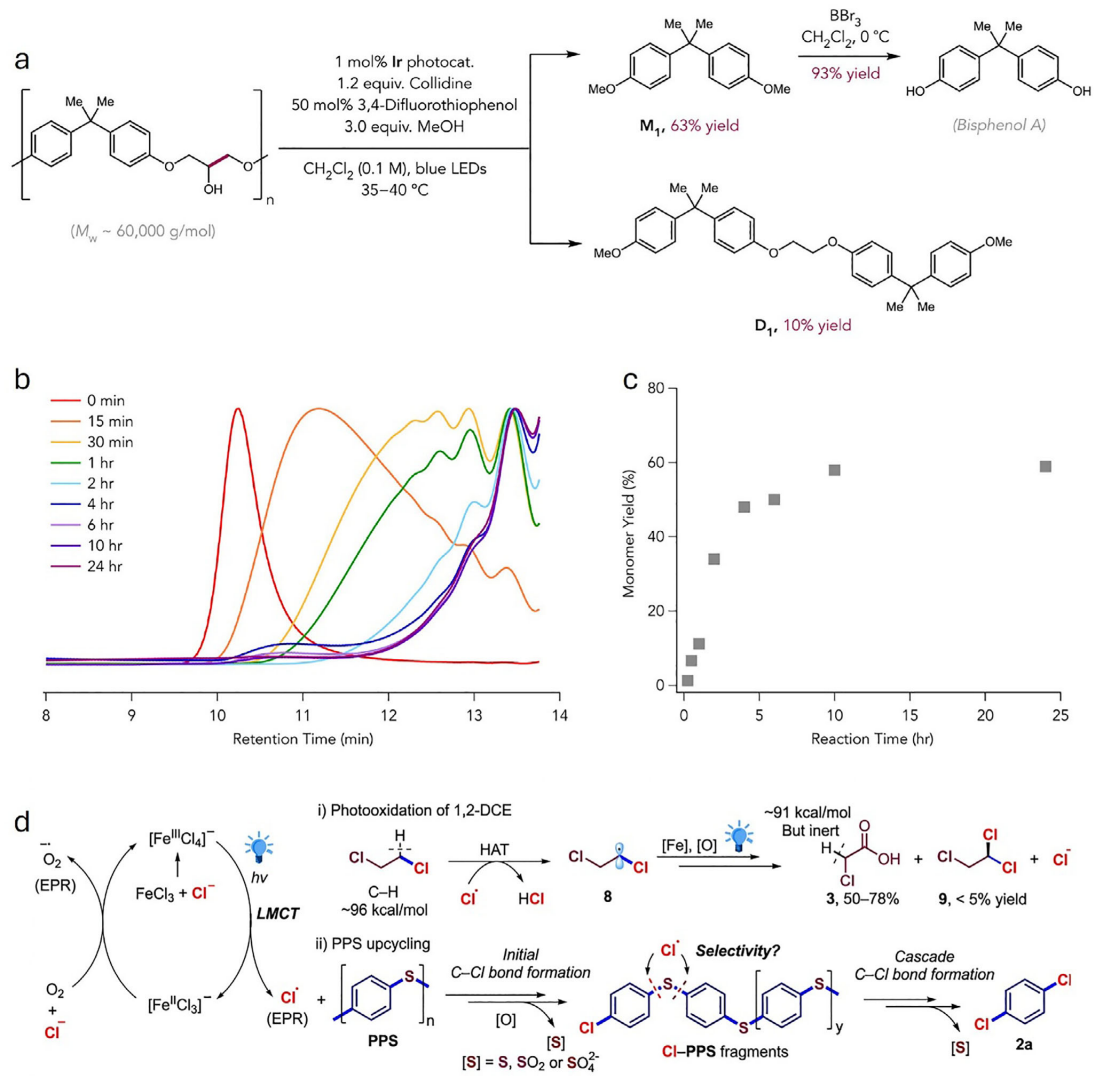
(a) Photocatalytic recycling of commercial phenoxy thermoplastic to BPA. Depolymerization reactions were performed on 0.5 mmol ‐OH (142 mg) scale. Yields were reported for isolated and purified materials and are the average of two experiments. (b,c) GPC traces and GC measurements showing the successful depolymerization of phenoxy and the increase in the GC yield of M1 over the course of 24 h. Reproduced with permission. Reproduced with permission [84]. American Chemical Society. (d) The proposed catalytic pathway. Reproduced with permission [53]. Wiley‐VCH.

PSFs and their related polymers are a class of plastics with outstanding chemical stability, resistance, and durability, thus these polymers are widely used. However, their recycling and upcycling remain highly challenging. A key challenge in upcycling of PSFs is how to selectively functionalize the inert aryl C‐SO_2_ (∼ 70–82 kcal/mol) or C─O bond cleavage in the polymer backbone [81, 82, 83]. Huang et al. reported an upcycling strategy for polyethersulfone (PES) plastics via photocatalysis, where CuCl_2_ served as the catalyst for the selective double desulfonylative chlorination of PES under ambient conditions, affording dichlorinated diaryl ethers (DDE) [54]. Using BASF's commercial PES resin as feedstock (Mn = 62.0 kg/mol, Mw/Mn = 1.40, where Mn is the number‐average molecular weight and Mw is the weight‐average molecular weight), a 67% yield of DDE was obtained. Upon addition of NaCl as an additive, the yield of DDE further increased to 85% after 48 h. Radical trapping experiments revealed the presence of trapped ∙Cl, ∙OH, and ∙O_2_^−^ radicals in the catalytic system, indicating that the reaction is driven by these three radical species. In this process, ∙Cl promoted the HAT process, while ∙OH and ∙O_2_^−^ facilitated cleavage of the polymer backbone. Mechanistic studies showed that the interaction between the copper salt and the PES chain can effectively promote conformational changes and structural disruption of the PES chain to activate the aryl sulfonyl units and induce the subsequent photo‐driven chain scission process. The aryl sulfone first underwent desulfonylative chlorination to generate DDE and an aryl sulfonyl chloride intermediate. Then the intermediate underwent a second desulfonylative chlorination under CuCl_2_ photocatalytic conditions to form another molecule of DDE. Nguyen et al. proposed PCET strategy for the depolymerization of a commercial phenoxy resin and high‐molecular‐weight hydroxylated polyolefin derivatives under visible‐light irradiation at near‐ambient temperature [84]. This reaction proceeded through activation of hydroxyl groups in the polymer backbone to generate highly reactive alkoxy radicals, which then undergo C─C bond β‐scission to promote chain cleavage. This process can effectively depolymerize phenoxy resin to afford the protected condensation monomer BPA dimethyl ether (BPADME, M_1_). Phenoxy resin with a Mw of approximately 60 000 g/mol was degraded using a catalytic system comprising 3 mol% Ir photocatalyst (Figure 7a). Under the optimized conditions, the monomeric product M_1_ was obtained in an average yield of 63%. Formation of a dimeric product, D_1_, was also observed, with an isolated yield of 10%, which was presumed to arise from radical‐radical homocoupling. Notably, M_1_ could be further demethylated with BBr_3_ to afford BPA in 93% isolated yield. The extent of phenoxy resin depolymerization as a function of time was subsequently investigated (Figure 7b,c). Gel permeation chromatography (GPC) results showed that the Mw of the polymer decreased markedly within the first hour of irradiation, accompanied by the appearance of lower‐molecular‐weight peaks. It was found that the polymer was essentially completely degraded after only 4 h, whereas the yield of M_1_ continued to increase over 10 h. These results indicate that, under photo‐driven PCET conditions, chain scission occurs rapidly, while the resulting oligomers are subsequently converted into the protected monomer through iterative C─C bond cleavage steps. This unexpected result was attributed to the instability of the target 2‐aryloxyacetaldehyde product under the polymer degradation conditions. To verify this hypothesis, the cleavage reaction of a phenoxy dimer model compound was examined under the same conditions, which was expected to generate an aldehyde product and a methoxybenzene‐containing product. If both products were stable, both should in principle have been obtained in quantitative yield. However, upon irradiation, cleavage of the dimer furnished the methoxybenzene‐containing product in 98% yield, whereas the aldehyde product was detected in only 21% yield, confirming that the latter was unstable under these conditions. It was proposed that decomposition of the 2‐aryloxyacetaldehyde proceeds via homolysis of the aldehydic C─H bond through a formal HAT event, thereby generating a formyl radical. This radical then undergoes decarbonylation, followed by hydrogen atom transfer from the thiophenol to ultimately afford the monomer M_1_. In addition, phenoxy resins are often blended with other polymers, their depolymerization behavior was further examined in the presence of several common plastics, including poly(vinyl chloride) (PVC, Mw ≈ 233 000 g/mol), PS (Mw ≈ 192 000 g/mol), and poly(methyl methacrylate) (PMMA, Mw ≈ 120 000 g/mol). Experimental results indicated that the presence of these polymers did not significantly affect phenoxy resin degradation, and the average yield of M_1_ remained in the range of 50–54%. GPC analysis of these hydroxyl‐free polymers before and after irradiation showed almost no change in either Mn, indicating that they did not undergo appreciable degradation under the PCET conditions. These results demonstrate a new polymer recycling strategy based on photo‐driven C─C bond cleavage, which may promote the development of next‐generation “degradation‐by‐design” polyolefin materials. PPS is a class of engineering plastics with excellent chemical resistance, thermal stability, and mechanical robustness, in which aromatic structures are interconnected through S atoms [85, 86]. A key challenge in the recovery of aromatic chemicals from PPS is the selective cleavage of the C─S bonds. Gu et al. reported a strategy for the upcycling of PPS at room temperature using 1,2‐dichloroethane and an FeCl_3_ photocatalyst, enabling the simultaneous production of the monomer 1,4‐dichlorobenzene (DCB, 2a) and high‐value product chloroacetic acid (CA) [53]. Experimental results showed that when FeCl_3_ was used at a loading of 10 mol%, the Mw decreased markedly from 27.1 to 2.6 kg/mol within 12 h, indicating extensive scission of the polymer chains, while the yield of DCB reached 82%. The authors subsequently applied the FeCl_3_ for upcycling of PPS resins and composites. PPS powder and pellet samples containing pigments and reinforced glass fibers all underwent dual desulfurative chlorination with 1,2‐dichloroethane under the optimized conditions, affording DCB in 60–82% yield and CA in 53–78% yield. Various post‐consumer PPS plastics, such as kitchenware and PPS films, were further investigated, and all of them afforded DCB after upcycling, with the highest yield reaching 89%, which further highlights the potential of this photocatalytic strategy for the valorization of PPS in mixed waste‐plastic streams. Mechanistic studies indicated that the reaction follows a radical‐mediated pathway, and the authors therefore proposed the catalytic pathway shown (Figure 7d). First, FeCl_3_ coordinated with chloride ions to form the active species [Fe^III^Cl_4_]^−^, which then undergoes ligand‐to‐metal charge transfer (LMCT) under light irradiation to generate ∙Cl and [Fe^II^Cl_3_]^−^. In the presence of chloride ions, [Fe^II^Cl_3_]^−^ is re‐oxidized by O_2_ to regenerate [Fe^III^Cl_4_]^−^, while simultaneously producing ∙O_2_^−^. The ∙Cl then abstracts a hydrogen atom from 1,2‐dichloroethane via HAT process to generate the 1,2‐dichloroethyl radical. This radical is subsequently oxidized to produce CA and chloride ions, or it may be trapped by a chlorine radical to form 1,1,2‐trichloroethane. Subsequently, the PPS chain reacts with ∙Cl via homolytic aromatic substitution (HAS) to generate Cl‐PPS fragments and PPS thiyl radicals. The PPS thiyl radicals are then rapidly converted into the corresponding Cl‐PPS fragments, with concomitant release of S‐containing species. However, during the upcycling of PPS plastics, the regioselectivity of chain cleavage in asymmetrically functionalized Cl‐PPS oligomers remained unclear. To address this issue, the authors employed DFT calculations to elucidate the subsequent reactions of asymmetric Cl‐PPS fragments. The results showed that, in asymmetric Cl‐PPS fragments, the more electron‐rich aryl C─S bond preferentially undergoes cleavage to ultimately form DCB. After the reaction, the Fe species could be recovered in 90% yield, while 78 vol% of the mixed solvent could also be recycled. This PPS upcycling study demonstrates that chlorinated phenylthio species are key intermediates. Through a ∙Cl‐mediated aryl C─S bond cleavage process, PPS depolymerization and the formation of functionalized PPS fragments can be achieved, providing useful guidance for the future upcycling of other C─S‐bond‐linked plastics.

## 6 Summary and Outlook

**FIGURE 8:**
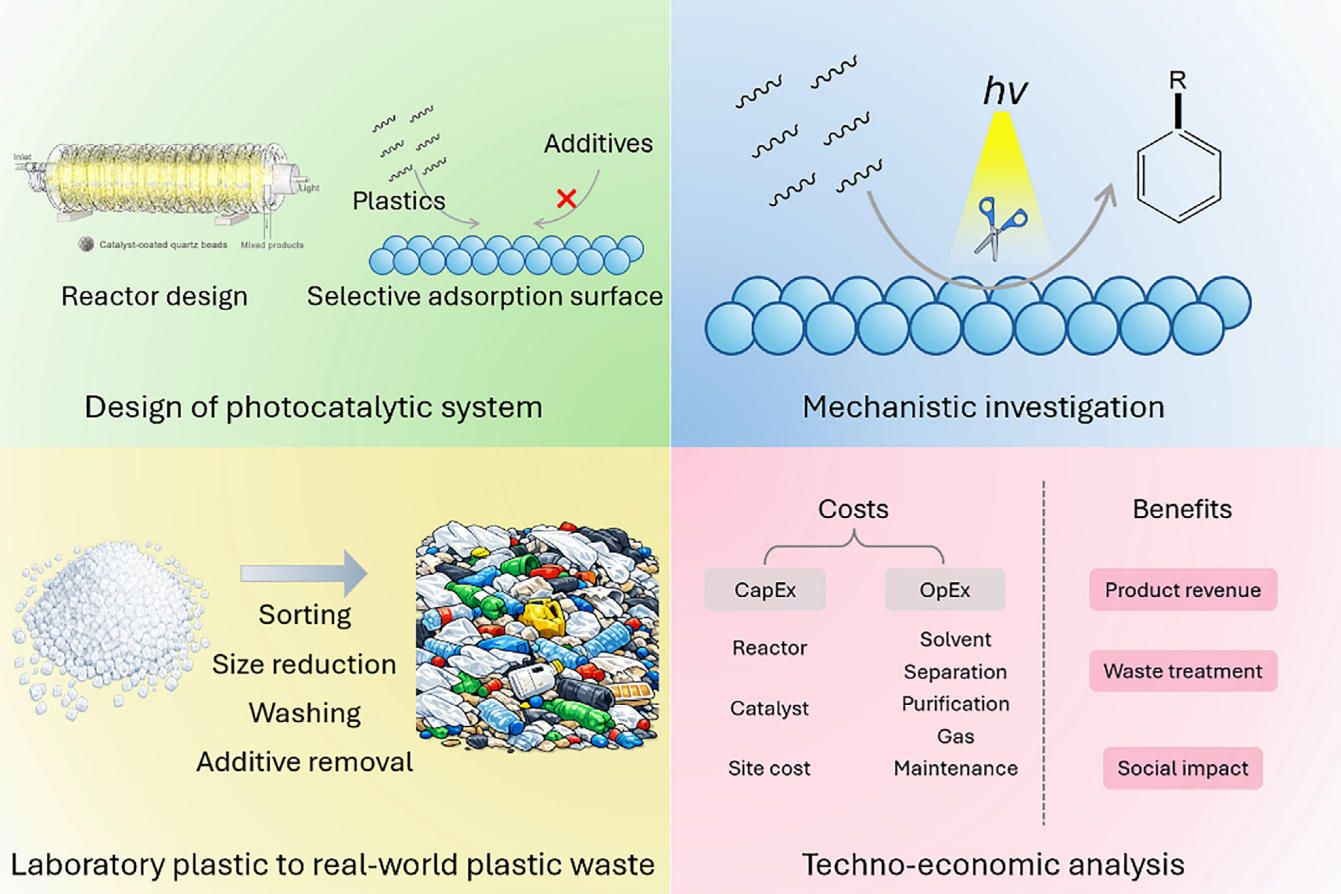
Schematic illustration of future perspectives.

Aromatic chemicals are mainly derived from petrochemical processes and are extensively utilized across diverse sectors [87]. The recovery of aromatic chemicals from waste plastics offers a promising strategy to reduce reliance on fossil resources while advancing resource circularity and sustainable development [42]. This review summarizes the latest advances in the photocatalytic recovery of aromatic chemicals from plastic waste, including the current mainstream catalysts, reaction pathways and mechanisms, and catalytic systems. PET, PS, PSF, PPS, and BPA‐based plastics contain abundant aromatic motifs, and extensive studies have explored their conversion into valuable aromatic chemicals. The photocatalytic recovery of aromatic chemicals mainly relies on radical‐driven processes, including ∙Cl, catalyst‐derived carboxyl radicals, and ROS. Radical‐mediated catalytic pathways can overcome the kinetic limitations associated with macromolecular chain segments, thereby facilitating chain scission. At present, the major aromatic products reported include BA (from PS), TPA (from PET), AP (from PS), and DDE (from PES). The main photocatalysts reported so far include metal chlorides (such as FeCl_3_, CeCl_3_) and organic carboxylic compounds, which have demonstrated excellent catalytic efficiency and high selectivity toward target products, promoting the upcycling of plastics and contributing to waste management and the development of a circular economy. However, photocatalytic plastic upcycling and chemicals recovery are still at an early stage of development. Catalyst stability and the separation efficiency of target products still need to be improved, while the recyclability of catalytic systems and their economic feasibility also require further attention. Therefore, the future development directions for photocatalytic plastic upcycling are proposed as follows (Figure 8):

1. Design and development of highly efficient, stable, and sustainable photocatalysis system. Photocatalytic systems for plastic upcycling generally comprise three key components: high‐performance photocatalysts, recyclable reaction solvents, separable products, and long‐term operation reactor. The integration of these elements is crucial for conversion of plastic waste into value‐added products and for practical applications [88, 89, 90]. At present, most reported efficient photocatalytic systems are homogeneous system, while the stability of most such photocatalysts remains below 50 h and still requires significant improvement. In contrast, heterogeneous photocatalytic system exhibits higher stability than homogeneous system, however, the catalytic efficiency is still challenging. So far, the types of photocatalysts reported for plastic conversion remain limited, such as TiO_2_, Fe_3_O_4_, CeO_2_, and CN_x_, and many other potential photocatalysts have yet to be explored. Heterogeneous photocatalytic systems mainly rely on ROS to drive catalytic reactions. Therefore, photocatalysts capable of generating ROS, particularly ∙O_2_^−^ and ^1^O_2_, deserve more attention. To address the challenges posed by additives, photocatalysts with selective adsorption capabilities could be designed. However, adsorption‐driven catalytic processes would face severe kinetic limitations associated with long polymer chains. Therefore, such catalysts may be more suitable for microplastic upcycling. In addition, high‐throughput screening strategies that combine experiments with simulations could enable the pre‐screening of catalysts and the identification of promising photocatalytic materials, thereby accelerating the development of high‐performance photocatalysts [91, 92]. The use of recyclable reaction solvents is also one of the key factors for the sustainability of photocatalytic plastic upcycling. For example, in PS upcycling systems, replacing dichloromethane with ethyl acetate can improve the sustainability of the catalytic system. Product separation efficiency is another critical factor that determines the practical applicability of plastic upcycling. The recovery of aromatic chemicals from plastics involves not only plastic depolymerization but also product separation. Separation efficiency directly affects the overall recovery efficiency. Furthermore, most photocatalytic systems rely on sealed batch reactors. Although these reactors are suitable for laboratory‐scale catalyst screening, they are not well suited for long‐term operation and online product separation. The practical implementation of photocatalytic recovery of chemicals from plastics requires the development of scalable reactor configurations. Among the emerging designs, spiral flow photoreactor have shown significant potential, which supports long‐term operation, facilitates online product separation, and offers controllable plastic degradation behaviors through adjustment of the solvent flow rate and O_2_ supply [93]. Accordingly, the development of efficient and sustainable catalytic systems requires the simultaneous consideration of all factors.
2. Mechanistic investigations. Photocatalytic plastic upcycling involves two major steps: cleavage of the polymer backbone and conversion of the resulting small molecular fragments into target products. Different plastic feedstocks require different bond scission during fragmentation process. For example, the upcycling of PS requires the cleavage of C─C bond, which involves C─H activation followed by β‐scission. The upcycling of PPS requires the selective abstraction of S atom and thus the cleavage of C─S bond. In addition, reactive species play a decisive role in driving the reaction. Therefore, identifying the dominant active species or the key driving force in reaction system is crucial for improving reaction efficiency and lowering the reaction barrier. Therefore, understanding reaction mechanism is essential for the design of highly efficient and selective photocatalytic systems for target‐product synthesis [67]. Moreover, product selectivity is closely related to the stability of intermediates in the catalytic system. Elucidating the reaction mechanism can regulate the adsorption and desorption behaviors of intermediates and products to optimize product selectivity. The combination of in situ characterization techniques, such as in situ EPR and in situ FTIR, can provide valuable insights into the main barriers associated with bond cleavage, the dominant reactive species responsible for driving the reaction, and the selectivity of key intermediates [94, 95]. Such investigations enable a deeper understanding of the catalytic process and mechanism, revealing the fundamental origins of product selectivity. Designing photocatalysts based on catalytic mechanisms is beneficial for achieving desirable catalytic performance. For instance, in PS upcycling, the major reaction barrier lies in C‐H activation. Introducing photocatalysts with strong hydrogen‐abstraction capability can facilitate C─H activation and thus improve catalytic performance [96, 97]. Therefore, mechanistic studies are important not only for understanding the reaction process itself, but also for guiding the rational design of catalytic systems.
3. Expansion of feedstocks from laboratory plastic models to real‐world plastic waste. The ultimate goal of photocatalytic plastic upcycling is to address the environmental pollution caused by plastic waste while recovering valuable chemicals from waste streams, thereby reducing reliance on petrochemical products [98, 99, 100]. Accordingly, the ultimate feedstocks for photocatalytic plastic upcycling should be real‐world plastic waste. However, the waste is often compositionally complex, contain a variety of additives, and are difficult to separate [101, 102]. And such additives can readily quench reactive radicals in the system. Therefore, addressing additive‐related issues is crucial when using real‐world plastic waste as feedstock. These additives can be categorized functional additives, colorants, fillers and reinforcements, including metals, metal oxides, volatile organic compounds (VOC), and oligomers [103]. Different types of additives can exert distinct effects on catalytic reactions. For example, metal oxides have little influence on photocatalytic recycling, whereas light‐stabilizing additives, such as hindered amine light stabilizers and hindered phenolic primary antioxidants, can specifically scavenge radicals and inhibit photodegradation. Therefore, classified recycling and pre‐sorting are particularly important. In addition, the removal or reduction of additives through pre‐treatment strategies can also mitigate their influence on subsequent catalytic processes. For additives with migratory characteristics, including antioxidants [104, 105], monomers and oligomers [106, 107], and light stabilizers [108], can be reduced through appropriate pre‐treatment strategies, such as solvent extraction, supercritical fluid extraction, dissolution‐precipitation, or microwave treatment [109]. For example, cyclic trimers and other low‐molecular‐weight oligomeric additives in PET can be removed by ∼ 60% after solvent extraction in xylene, DCM, acetone, water, and hexane at 140°C for 24 h. With the assistance of microwave treatment, comparable removal efficiency can be achieved within a much shorter time of 30–120 min [110]. Similarly, ∼70% of flame‐retardant additives in PS can be removed after solvent extraction in toluene at 110°C for 6 h. However, as the molecular weight of the additives increases, the extraction efficiency decreases. Moreover, for some stabilizers or compatibilizers, their specific properties can be utilized to promote catalytic reactions [111]. For example, PVC readily undergoes HCl elimination and forms unsaturated chains during recycling and reprocessing. Therefore, thermal stabilizers are commonly added to improve its processing stability. Retaining these stabilizers during PVC recycling can effectively suppress HCl elimination. Similarly, in the recovery of aromatic chemicals, retaining analogous stabilizers may help prevent the cleavage or loss of aromatic moieties. Retaining compatibilizers may also facilitate plastic dissolution in solvents, thereby reducing the complexity of the solvent system. Beyond additive management, it is necessary to develop long‐lived radical systems or poisoning‐resistant photocatalysts. Real‐world plastic waste usually consists of mixtures of different plastics, making it highly challenging to develop efficient catalytic systems applicable to diverse plastic substrates. In a reported catalytic system based on FeCl_3_ and NH_4_Cl in a HEIP medium, the strong oxidizing nature of ∙Cl enabled the recovery of TPA from four types of PET bottles. The introduction of radical systems with broad applicability and strong oxidative capability may therefore enable the depolymerization of various plastic wastes and promote the upcycling of real‐world plastics. In addition, the design of multi‐electron transfer pathways with reduced reliance on radical‐mediated processes, or adsorption‐dominated catalytic systems enabled by catalyst surfaces with selective adsorption capability, represents a promising direction. However, such catalytic processes are often highly sensitive to mass‐transfer limitations. Therefore, these catalysts may be particularly suitable for microplastic recycling, or alternatively, plastic waste should undergo crushing pre‐treatment to enhance catalyst‐substrate contact before recycling.
4. Development of techno‐economic analysis (TEA). The practical implementation of photocatalytic plastic upcycling requires consideration of multiple factors associated with the transition from laboratory‐scale studies to industrial application [112, 113, 114]. Therefore, an appropriate TEA is essential. Rather than merely cataloguing cost items, key economic parameters, particularly capital expenditure (CapEx) and operating expenditure (OpEx), should be systematically evaluated to identify the major cost drivers of the process. For photocatalytic plastic upcycling, CapEx primarily includes the costs of reactors and catalysts, and the reactor footprint, whereas OpEx mainly comprises solvent and gas consumption, product separation and purification, and equipment maintenance. In addition, the costs associated with additive separation should also be considered to OpEx. Where feed pretreatment is necessary, the economic impact of solvent‐based and thermal treatment, together with the management of waste solvents and gaseous emissions (e.g., CO_2_ or CH_4_), should be considered to OpEx. On the benefit side, process viability will depend on both the market value of the target products and the potential economic advantages of using plastic waste as low‐cost feedstocks. In addition, the broader social benefits of waste treatment should also be taken into account. Although no dedicated TEA has yet been reported for photocatalytic plastic upcycling, these key parameters can provide guidance for the TEA of photocatalytic plastic upcycling.

## Author Contributions

**Jun Huang**: Writing – review and editing. **Jutarat Jitrada**: investigation. **Lizhen Liu**: writing – original draft, writing – review and editing, conceptualization.

## Funding

The authors acknowledge the financial support from Australian Research Council Discovery Projects (DP250104311), Future Fellow Project (FT220100601), and Linkage Project (LP200200615), the Sydney Nano Grand Challenge Project, and NSW Decarbonisation Innovation Hub Powerfuels including Hydrogen Network.

## Conflicts of Interest

The authors declare no conflict of interest.

## Data Availability

The data that support the findings of this study are available from the corresponding author upon reasonable request.
